# Cell-Penetrating and Targeted Peptides Delivery Systems as Potential Pharmaceutical Carriers for Enhanced Delivery across the Blood–Brain Barrier (BBB)

**DOI:** 10.3390/pharmaceutics15071999

**Published:** 2023-07-21

**Authors:** Soma Mondal Ghorai, Auroni Deep, Devanshi Magoo, Chetna Gupta, Nikesh Gupta

**Affiliations:** 1 Department of Zoology, Hindu College, University of Delhi, Delhi 110007, India; 2 Department of Chemistry, Hindu College, University of Delhi, Delhi 110007, India; 3 Department of Chemistry, Hansraj College, University of Delhi, Delhi 110007, India; 4 Pharmaceutical Sciences Division, School of Pharmacy, University of Wisconsin-Madison, WI 53705, USA

**Keywords:** cell-penetrating peptides (CPPs), endosomal entrapment, homing peptides, blood–brain barrier (BBB), tumour-specific markers, siRNA-CPP delivery

## Abstract

Among the challenges to the 21st-century health care industry, one that demands special mention is the transport of drugs/active pharmaceutical agents across the blood–brain barrier (BBB). The epithelial-like tight junctions within the brain capillary endothelium hinder the uptake of most pharmaceutical agents. With an aim to understand more deeply the intricacies of cell-penetrating and targeted peptides as a powerful tool for desirable biological activity, we provide a critical review of both CPP and homing/targeted peptides as intracellular drug delivery agents, especially across the blood–brain barrier (BBB). Two main peptides have been discussed to understand intracellular drug delivery; first is the cell-penetrating peptides (CPPs) for the targeted delivery of compounds of interest (primarily peptides and nucleic acids) and second is the family of homing peptides, which specifically targets cells/tissues based on their overexpression of tumour-specific markers and are thus at the heart of cancer research. These small, amphipathic molecules demonstrate specific physical and chemical modifications aimed at increased ease of cellular internalisation. Because only a limited number of drug molecules can bypass the blood–brain barrier by free diffusion, it is essential to explore all aspects of CPPs that can be exploited for crossing this barrier. Considering siRNAs that can be designed against any target RNA, marking such molecules with high therapeutic potential, we present a synopsis of the studies on synthetic siRNA-based therapeutics using CPPs and homing peptides drugs that can emerge as potential drug-delivery systems as an upcoming requirement in the world of pharma- and nutraceuticals.

## 1. Introduction

There are considerable breakthrough discoveries on the structure and development of synthetic and natural drugs that have gained importance in science and clinical studies. Often, scientists from around the globe create compounds that possess the potential to revolutionise health sciences. However, not all of them produce significant results when administered. Brain tumours and associated cancers are often fatal to patients because treatment such as chemotherapy is complicated due to extensive intratumoral heterogeneity that renders poor penetration of the drugs through the blood–brain barrier (BBB), causing less bioavailability of drugs and a lack of selective tumour targeting [1]. Thus, the success of any drug requires a comprehensive analysis of its mode of action as well as a study of the system targeted by the compound of interest.

**Figure 1:**
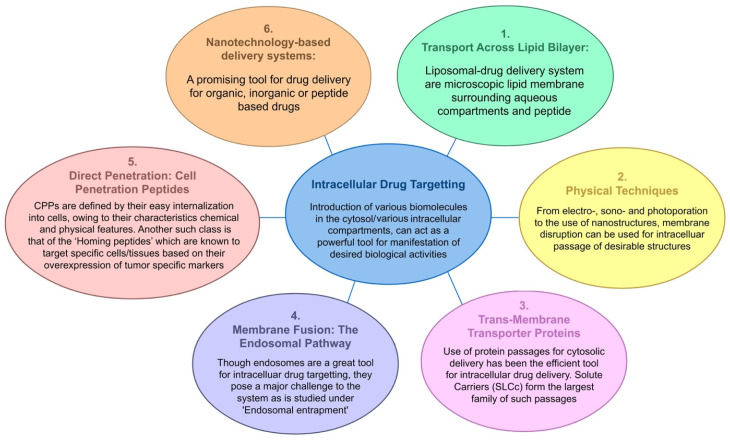
Intracellular drug targeting and the various drug-delivery pathways (the figure has been prepared using Adobe Animate CC software, https://www.adobe.com/in/products/animate.html (accessed on 24 June 2023)).

A very superficial drug classification involves two terms in daily use: (i) extracellular and (ii) intracellular drug delivery. The latter has captured the attention of medicine because the introduction of various biomolecules into the cytosol (or a targeted intracellular compartment) is a powerful tool for the manifestation of desired biological activities. For a long time, transport across the lipid bilayer remained the major challenge. Micelles provided a solution to this long-encountered transportation hurdle as they could transport a series of different molecules ranging from hydrophobic drugs and proteins to genes [2]. Transport systems soon became increasingly sophisticated, and today we are the proud possessors of multiple techniques that make targeted drug delivery feasible (Figure 1). Such techniques can be conveniently identified as macro, micro, or nano techniques based on their impact resolution [3]. The passage of compounds across the plasma membrane can be obtained by membrane fusion, endocytic pathways, trans-membrane transporter proteins, and membrane-disruption-facilitated techniques such as direct cell penetration or increased permeability [4]. The last decade has witnessed a plethora of the latest techniques enabling small cell-penetrating peptides (CPPs), peptide shuttles, and brain-permeable peptide–drug conjugates (PDCs) to cross the formidable barrier of the brain parenchyma and endothelial cells. In order to achieve a delayed-release pattern, it is essential that our drugs of interest be encapsulated within biocompatible carriers that increase their plasma life and stability. As a result, peptide-based drug delivery systems have an advantage over current medications [5]. One such mechanism of intracellular drug delivery brings CPPs into the picture. Furthermore, most circulating physiological ligands are either proteins/peptides or peptide-conjugated complexes; thus, peptides are also considered the better choice for specific or targeted drug delivery.

Despite the number of advantages that this system possesses, one cannot completely rule out the advantages of the lipid-mediated mechanism, which practically started the science of intracellular molecular delivery [2]. Hence, lipid–peptide conjugates have arisen as the new mediators of nutraceuticals, with increased biological stability and mechanical strength, controlled release, greater circulation time, targeted delivery, and decreased cytotoxicity [4,6]. Moreover, passage through the lipid bilayer demands optimal hydrophobicity. These distinct structural features have rendered CPPs as potential molecules in the science of drug (both biomolecule and nanoparticle) delivery. TAT-peptides conjugated to iron oxide nanoparticles were the first to be used for CPP-mediated nanoparticle delivery across the BBB [7]. Similarly, solid-lipid nanoparticles (SLNs) conjugated to TAT-peptides have been delivered to CNS without compromising the integrity of the BBB [8]. CPP-modified quantum dot-loaded polymeric micelles were prepared from a copolymer polyethylene glycol phosphatidyl ethanolamine (PEG–PE) bearing the TAT–PEG–PE linker and have been the most used CPPs for therapeutic delivery across the BBB [9].

Additional classes of targeting peptides are the ‘homing peptides’. These classes of peptides have become helpful in cancer research, especially for brain tumours, as they are known to target specific cells/tissues based on their overexpression of tumour-antigens or specific markers [10]. This article focuses on peptide-based delivery systems and the implications of various ways of reaching tissues by penetrating the blood–brain barrier.

## 2. Cell-Penetrating Peptides (CPPs)

Two decades ago, the concept of peptide-transduction domains (PTDs) emerged with the observation of transcription factors that could move to and from the cell membrane as well as from one cell to another [11]. The 1988 discovery is credited to Frankel and Pabo, who demonstrated that the HIV-1 Tat (transcription-transactivating) protein not only enters the cells but also relocates to the nucleus [12]. A series of such observations, studies, and discoveries finally led to the era wherein the PTDs of CPPs can deliver drugs/medication(s) into cells of interest. Simply, CPPs can be considered the hitchhiker’s ride to a predetermined destination. The transport can take place either by covalent bonding, leading to the formation of a drug–CPP conjugate [13], or by the formation of non-covalent conjugates [11]. CPPs are essentially a 15–25 long amino-acid sequence of amphipathic molecules rich in positively charged amino acids, primarily arginine. Arginine is preferred over lysine owing to the extra H-bond of the guanidium group. Naturally, all characteristic features of CPPs are primarily aimed at improving internalisation into the cells.

The Pep- and MPG families of small peptides are instances of such amphipathic cell-penetrating molecules that can form conjugates with proteins and nucleic acids, respectively, and can aid in obtaining the desired results [14]. CPPs can form peptide nucleic acid (PNA) conjugates, which can increase the uptake of therapeutic nucleic acids by cells of interest. This increase in PNA uptake by hepatocytes was studied by Ndeboko et al. to inhibit replication in duck hepatitis-B virus following a low-dose administration [15].

### 2.1. Various Strategies of CPP-Mediated Drug Delivery

CPPs are designed to successfully deliver macromolecules into the cytosol; thus, they are used as delivery systems rather than therapeutic agents [16]. CPPs may be transported directly across the cellular membrane or by entrapment as peptides/cargo within the endosomes. Endocytic pathways usually involve one of the energy-dependent mechanisms such as phagocytosis, caveolae-mediated endocytosis (CvME), clathrin-mediated endocytosis (CME), or cholesterol-dependent endocytosis. In the uptake of peptides like TAT, polyarginine, and NickFect families of peptides (NF51/NF1), it was observed that macropinosomes are formed by rearrangement of actinic cytoskeletal elements and invagination of the cellular membrane, thus entrapping extracellular fluid [17,18]. Similarly, reordering the actinic cytoskeletal elements by clustering the caveolin-1 proteins was used for the uptake of CPPs with cargoes such as p18, p28 azurin fragment, CVP1 (chicken anaemia-derived CPP), PepFect14/DNA conjugate, and TAT via the CvME pathway [19,20]. In clathrin-mediated endocytosis (CME), the interaction of peptides with specific cell surface receptors leads to the formation of vesicles in phosphatidylinositol 4,5-biphosphate-rich regions of the plasma membrane. Thereafter, an adaptor protein binds to phosphatidylinositol 4,5-biphosphate forming coated pits where dynamin, the energy-rich GTPase, cleaves and releases the clathrin-coated vesicles with their delivery to early endosomes [21,22]. Anionic CPPs, oligo-arginine, and TAT are known to involve CME in peptide delivery to the cells [23].

**Figure 2:**
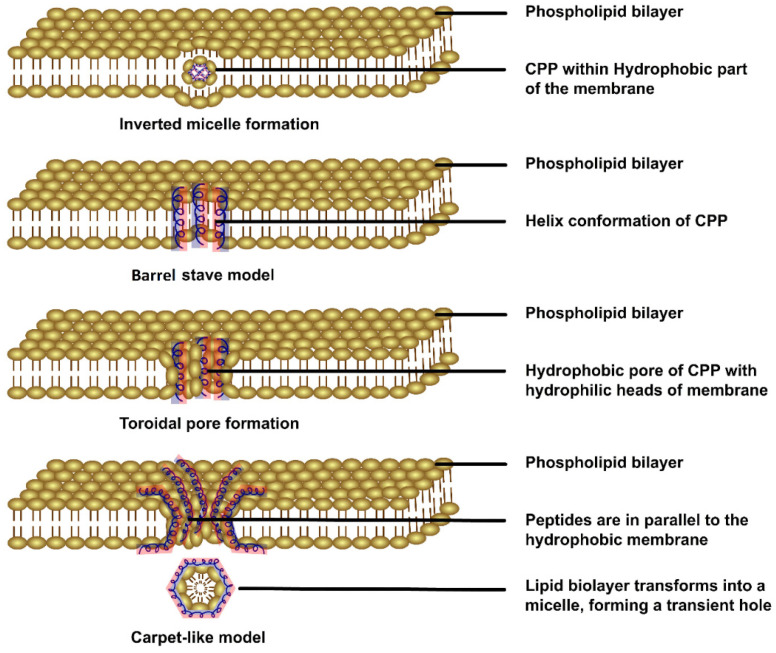
Graphical representation of the direct internalisation of CPPs via cell membranes. The blue and red colours represent the hydrophobic and hydrophilic parts of the peptide, respectively [25] (the figure has been created using Adobe Animate CC software, https://www.adobe.com/in/products/animate.html (accessed on 25 June 2023)).

At physiologically low temperatures, when a positively charged CPP interacts with the negatively charged phospholipid bilayer of a membrane, it may skip adherence to the lipid bilayer and be translocated without the aid of energy [24]. This direct translocation involves four different internalisation methods, namely, the inverted micelle model, barrel stave model, carpet-like model, and toroidal pore model [25]. In the inverted micelle model, conjugated hydrophilic CPPs interact with hydrophobic inner lipid membranes, forming hexagonal micelles that release the cargo after interaction with the inner membrane, thereby destabilising the micelle [19]. At a high concentration of CPPs and high pH, perpendicular pores are formed on the cell surfaces lined by hydrophilic residues of the CPP encircling the internal milieu of the pores; this is known as the barrel stave model [24]. Böhmová et al. proposed the toroidal pore model and carpet-like model for direct translocation [22]. In the toroidal model, the hydrophilic residues of CPPs are associated with the polar lipid heads, forming a wall that houses both the inserted peptides within the hydrophilic phospholipid cell membrane and, in the carpet model, this interaction leads to remodelling of the cellular membrane as internalisation occurs without the hydrophobic core, forming a hole in the membrane (Figure 2).

### 2.2. Escape of CPPs from Endosomal Entrapment and Protease Degradation

CPPs at low levels are classically internalised via endocytic pathways, and the macromolecules used as therapeutic peptides often enter cells and become entrapped inside endosomes. Endosomal escape represents a major hurdle for the usage of CPPs as delivery systems and/or therapeutics [26,27]. The endosome escape route for CPPs is difficult to envisage and still not fully understood [28,29]. However, the endosomal escape of CPPs can be achieved if they are translocated in the cytosol where the therapeutic targets are situated without disturbing the endosomal membranes or causing toxicity. In this section of the manuscript, we present the many strategies mentioned in the literature as well as some future directions that indicate the mechanisms by which CPPs can escape endosomal entrapment.

Cell toxicity caused by strong peptide–lipid interactions may be harmful to the cell but may help the CPP to escape the endosomal membrane. Thus, key properties may be harnessed for the selective benefit of CPPs. Endosomes undergo the high synthesis of bis(monoacylglycerol)phosphate (BMP) in the late phase, which makes them more acidic on maturation. This shift in pH driven by a proton pump facilitates some CPPs to undergo alterations in their three-dimensional structure and helps them to cross endosomal membranes [30,31,32].

In some studies, it has been shown that a six-polyethylene glycol unit (PEG-P6-GFWFG) TAT used as a CPP, in combination with hydrophobic endosomal escape domains (EEDs), significantly downregulated cellular toxicity while sustaining cell-penetrating capabilities [33]. Histidine residues with the imidazole group changed to positively charged motifs at a pH below 6; hence, poly-histidine sequences contribute to endosomal escape [34]. This was successfully demonstrated in delivering plasmid DNA with the reporter protein luciferase to human glioma cells in the brain using TAT, covalently attached to 10 His residues (TAT10H) [35].

In 2014, Qian et al. proposed that the cyclic peptide cFΦR4, commonly used for stability, could be strategically internalised through endocytosis, and thereafter escape from endosomes [36]. Along similar lines, oligomerisation of CPPs is also considered a resourceful approach to counter endosomal entrapment. A CPP TAT (dfTAT) was designed to form dimers between two Cys residues, which enhanced cytosolic release by 90-fold, whereas its earlier efficiency was only 1% [37]. This suggested that these peptides reach the cytosol via endocytosis and escape because of pH acidification [38]. Moreover, chirality improved the stability and penetration ability of D-dfTAT. This peptide also showed better resistance to protease digestion and enhanced the lytic ability of the endosome membrane.

**Figure 3:**
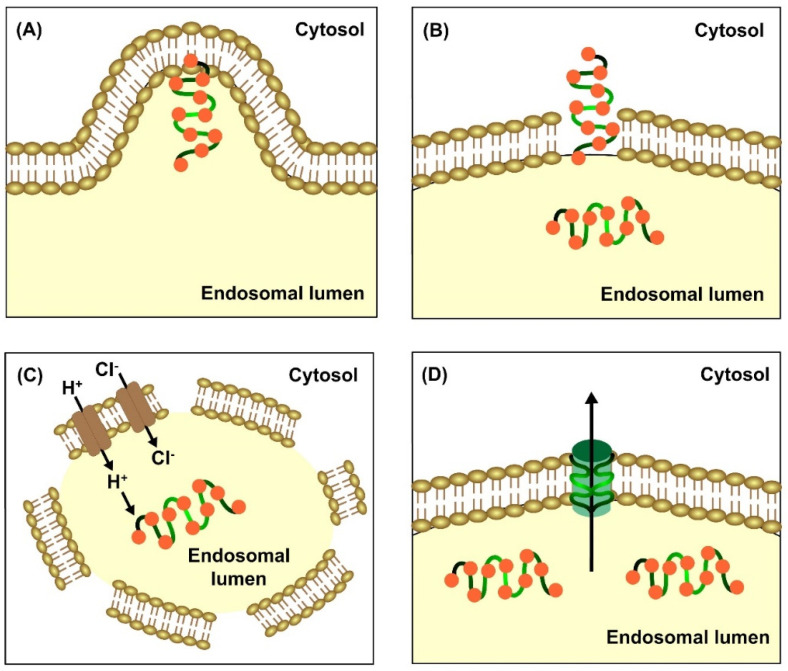
Various mechanisms to escape endosomal entrapment include (**A**) budding, (**B**) membrane disruption, (**C**) proton sponge effect, and (**D**) pore formation (the figure has been created using Adobe Animate CC software, https://www.adobe.com/in/products/animate.html (accessed on 24 June 2023)).

The CPPs that can successfully overcome endosomal entrapment usually employ the following mechanisms to escape endosomes: (a) Budding: This proposal has recently gained prominence as it is very likely that high concentrations of CPPs can lead to the formation of smaller vesicles by cutting off from the original endosome that readily degrades and releases their content [39]. (b) Membrane disruption: CPPs are known to possess positively charged surfaces that tend to react with negatively charged acyl chains of phospholipid headgroups of the lipid bilayer. This causes transient disruption of the endosomal membrane and the release of cargoes [40,41]. (c) Proton sponge effect: The sustained influx of protons into the luminal space of endosomes causes the internalisation of chloride ions, which leads to the osmotic imbalance and mechanical disintegration of endosomes [42,43]. Of late, studies have claimed the proton sponge hypothesis to be non-feasible and unrealistic [44,45]. (d) Pore formation: Considering CPPs as bacterial endotoxins, they behave similarly by inserting and oligomerising into the lipid bilayer. The hydrophobic cores of CPPs form defined pores that make the endosomal membrane permeable to release the inside content [46,47]. This theory has also been challenged as few macromolecules are delivered into the cytosol that are larger than the pore diameter created by peptide oligomerisation [48]. Mechanisms to escape endosomal entrapment is illustrated in Figure 3A–D.

Clearly, there is no ‘rule of thumb’ to overcome this challenge of endosomal entrapment. It is an uphill task to achieve a win–win situation of not breaking the cell membrane while breaking free from the endosomal membrane. To achieve endosomal selectivity, several factors that affect cellular uptake and translocation across the plasma membrane should be employed. The uptake of cargo depends first on the composition of the lipid and protein content of the plasma membrane and second on the concentration and physiochemical properties of the peptide and its cargo. CPPs with a high positive charge, e.g., more arginine domain with the guanidinium group as well as amphipathic peptides, are better suited for direct cellular uptake than endocytosis. Generally, at high concentrations, direct transportation occurs by temporarily destabilising the plasma membrane and, at low concentrations, endocytosis is observed. Transportan, a primary amphipathic CPP, and arginine-rich CPPs at low concentrations are mainly endocytosed, while rapid cytoplasm entry occurs at higher concentrations. Similarly, at higher concentrations, CPPs like R8, R9, and TAT are taken up via vesicular structures like clathrin or endosomes and, at low concentrations, uptake mainly occurs via nonendocytic nucleation zones or direct transportation across the plasma membrane. But there are many alterations to these observations. Penetratin at low concentrations leads to direct translocation, while at high quantities, endocytosis prevails [19]. Thus, CPP uptake, depending on concentration, can be more complex than envisaged.

A recent paper by Nadal-Bufí et al. presented the strategies that form the basis of future directions towards disabling the endosomal entrapment of CPPs and releasing therapeutic peptides to their targeted site [49]. First, CPPs should be designed to deliver therapeutic peptides by the optimisation of EEDs or the identification of new EED sequences. EEDs characteristically have cationic and hydrophobic residues that can selectively bind and disrupt cell membranes and reduce toxicity. Among the natural sources, virus and antimicrobial peptides possess high lytic activity and active membrane properties; thus, they can be employed to design EEDs. Also, at an acidic pH, the overall positive charge of CPPs is enhanced, which improves cellular uptake and endosomal escape. Incorporating Arg-residues within the sequence of CPPs can be an approach to increase the positive charge against endosome membranes with a high proportion of negatively charged lipids. Thus, cyclisation of stereochemical changes in CPPs can increase their uptake as well as improve endosomal escape [50]. However, there is no certainty in some CPPs that have the capacity to permeabilise and escape from endosomal membranes to reach the cytosol [51]. Moreover, sometimes the cargo itself can change the properties of CPPs; thus, a sound understanding is required to judiciously strategise therapeutic CPPs that can target intracellular proteins as well as escape endosomal entrapment.

Targeting peptides face another problem of proteolytic degradation within the cellular compartments. Nanomaterial-based drug delivery systems have been proven to cross the BBB either by carrier-mediated transcytosis (CMT), adsorptive-mediated transcytosis (AMT), or receptor-mediated transcytosis (RMT) and have often offered protection to proteolysis. Lipid-based nanoparticles (NPs) with surface modifications via transferrin, lactoferrin, glucose, and glutathione polyethylene (PEG) are more effective in BBB permeability. PEGylation of gold and silica NPs have also been shown to increase biocompatibility. Polymeric-based NPs such as chitosan, hydroxyl polyamidoamine (PAMAM), and poly (D,L-lactide-co-glycolide) (PLGA) have better physical and chemical properties and are highly resistant to degradation [52].

The modification of peptide sequences including amino acid incorporation within the backbone or the non-canonical side chains, enantio/retro-enantio isomerisation, and the cyclisation of N and C-termini further enhances protease resistance. Peptides modified with such changes are termed ‘peptidomimetics’. N and C termini modifications prevent exoprotease-mediated hydrolysis. Backbone changes like isosteric replacement of amide bonds, carbon skeleton extension or amide alkylation, N-methylation and α-methylation, or the addition of β or γ amino acid residues impart protection from endoproteases. These changes increase lipophilicity by reducing hydrogen bond formation, thereby enabling peptides to cross biological barriers. The D-enantiomeric amino acid is usually a ‘retro-enantio isomer’ that displays side chain topology like that of its native L-form with inverted amide bonds. Such retro-enantio isomers have reduced immunogenicity and are resistant to proteolytic degradation. Cyclical peptides have better biological activity than linear peptides as the cyclical configuration is mostly favoured for high peptide affinity due to better binding of the target protein [53].

## 3. Homing Peptides for Targeted Drug Delivery

One class of tumour-homing peptides (THPs) includes 3–15 residues and long, receptor-specific peptide molecules wherein the target receptors can vary from intracellular to cell-surface bound receptor molecules [10]. THPs can be characterised and identified based on specific sequences that can recognise and bind to receptors widely expressed in tumour cells. Integrins are among such commonly recognised cell-surface receptors. These receptors play a primary role in anchorage by binding cells to the extra-cellular matrix. These integrins can identify short peptide sequences or tripeptides like Arg-Gly-Asp (RGD) [54]. This RGD peptide was the first to be documented against endothelial cell integrins [55]. Such peptides are known to form many drug conjugates, thus easing the process of drug delivery owing to their strikingly small sizes and low molecular weights. Another similar tripeptide, NGR (Asn-Gly-Arg), is known to target the endothelial cells of neoangiogenic vessels [56]. To bring our drugs from paper to practice, these small peptides can conveniently act as vehicles for targeted drug delivery. Hence, these peptides are now at the heart of advanced cancer medicine and associated research.

Unlike the CPPs that can be internalised by diverse cell lineages, THPs show receptor-mediated, endocytic cellular internalisation and are hence increasingly relevant for lineage- or tumour-specific drug targeting [57]. Evidently, in addition to chemical composition, specificity, mode of action, and physiological impacts, another essential criterion for drug selection is the time of action. Certain conditions might demand delayed or sustained drug release while others might call for an immediate or burst release. Most SOS or over-the-counter medications should ideally belong to the latter class. Both CPPs and THPs can be manipulated for temporally monitored administration of drugs. This conclusion can be easily drawn because the collective process of THP binding and incorporation into a target cell requires less than 120 min [57]. A series of studies on colon-cancer-homing peptides (CPP2) and myeloid-leukaemia-homing peptides (CPP44) brought us to believe that these homing peptides are taken up in an ATP-dependent manner and that their internalisation is not influenced by serum components. In addition, certain CPPs such as CPP44 show selective and preferential entry in tumour cell lines only [58]. This selectivity can be a tool in minimising or altogether negating the adverse physiological impacts of cancer medications and therapies.

## 4. Peptide-Mediated Drug Delivery Systems across the Blood–Brain Barrier

### 4.1. Introduction to the Blood–Brain Barrier (BBB)

The brain, like all other vital organs of the body, needs nutrients and gases to function properly. It is substantially protected by three coatings of meninges protecting the BBB from overexposure to potassium, glutamate, and glycine, which, at increased concentrations, can be neurotoxic [59]. Armed with a widespread blood capillary network, the BBB is considered an important barrier that regulates drug molecule access to the brain parenchyma. Tight junctions (TJs) and adherens junctions (AJs) are the two main junctional complexes of the BBB that regulate the influx and efflux of substances through the paracellular pathway connecting the endothelial cells of brain capillaries. Apart from the BBB, the blood–cerebrospinal fluid barrier (BCSFB), circumventricular organ barrier (CVOB), and arachnoid barrier (AB) filter out small and large drug molecules and 98% of pharmaceuticals [60]. In most cases, it has been noted that most drugs remain inaccessible to the brain as they are flushed out by the BBB via the return journey of the CSF to the blood or through the transporters present in the brain parenchymal cells [61]. The extracellular base membrane, a layer of endothelial cells (ECs) connected to astrocytes (ACs) and pericytes (PCs), and microglia form the neurovascular units (NVUs) of the BBB, which stops the penetration of drugs into the CNS [62,63]. Drugs administered via intravenous routes are unable to cross NVUs, which has remained a challenge to date [64].

**Figure 4:**
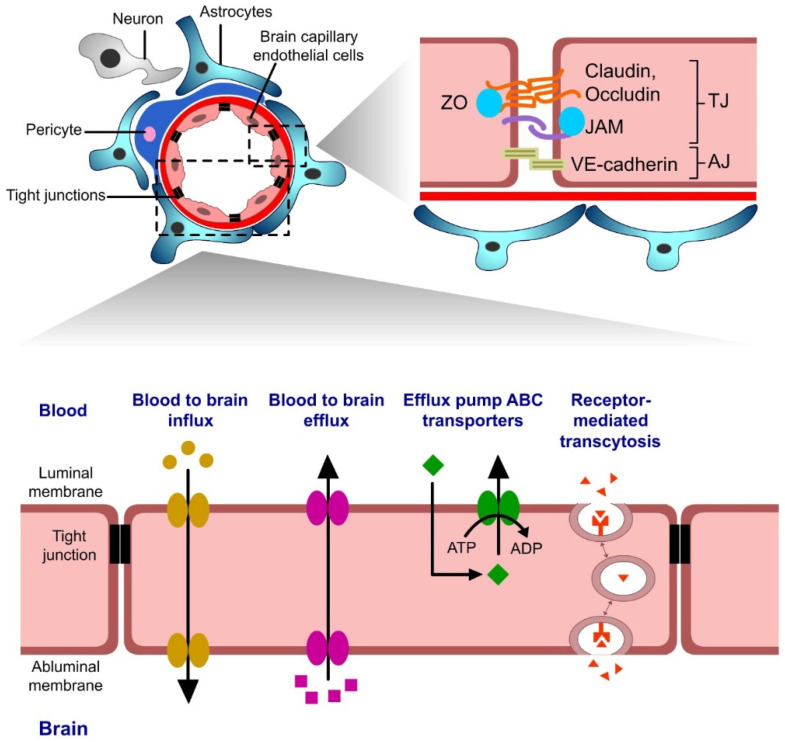
Various cellular interactions and the transport across the blood–brain barrier (the figure has been created using Adobe Animate CC software, https://www.adobe.com/in/products/animate.html (accessed on 24 June 2023)) [66,69].

Several ways of transport are known that enable drug molecules, lipid-soluble small molecules, weak bases, and electrically neutral solutes to diffuse passively across the BBB, as shown in (Figure 4) [65,66]. Any drug molecules that can passively diffuse across the BBB should have a molecular weight of less than 400 Da, good lipophilicity, a log of the octanol–water partition coefficient (logPo/w) between five and six, and fewer than eight hydrogen bonding groups [67,68]. This passive diffusion may transfer nutrients/drugs by passing through the intracellular space (paracellular) or moving solutes through a cell (transcellular). Regrettably, it has been determined that more than 98% of drugs targeted to CNS cannot cross the BBB at the minimum therapeutic concentration [18]. Thus, CPPs and homing peptides are new strategies anticipated to escape the BBB, thereby improving drug delivery to the CNS [59].

### 4.2. Cell-Penetrating Peptides as Delivery Systems across the Blood–Brain Barrier

CPPs are the peptide-based drug delivery system that holds promising and attainable prospects to deliver drugs to the brain. These small synthetic peptide shuttles (containing natural amino acids) enable the influx of a varied range of small molecules across the BBB. These natural peptides are derived from various sources, namely, HIV proteins (TAT, RI-OR2-TAT), the rabies virus (RVG-29), phage receptors (Pep-22, TGN, G23, T7, THR), venom neurotoxins (Apanin, MinApa4), and neurotropic endogenous peptides (regulon polypeptides, RAP, angiopep-2). Incidentally, although these compounds are highly pathogenic or toxic, they are reported to be non-toxic to neuronal cells [70].

**Figure 5:**
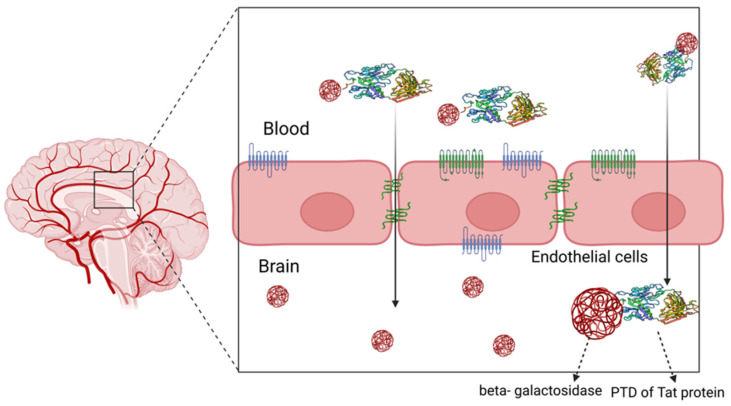
Drugs conjugated to Tat-protein can be targeted across the blood–brain barrier. CPPs can show transcellular movements either by traversing across junctions or through endocytic pathways (the figure has been created with the help of BioRender).

Despite the well-studied ability of CPPs to enter mammalian cells, it is only a few fragmentary studies that mention their transcellular aspects [71]. A study conducted on Caco-2, the human colon cancer cell line, investigated the differential penetration of three different CPPs across the plasma membrane, namely, transportan, penetratin, and TP-10, and it was concluded that Transportan and transportan analogue TP-10 traverse the membrane primarily by a transcellular mechanism [72]. Similar studies conducted for Tat proteins showed a plasma–membrane permeation barrier in well-differentiated epithelial cell lines, i.e., Caco-2 and MDCK, which was absent in HeLa [73]. A BBB transport study conducted for such peptides demonstrated wavering levels of cell penetration wherein the Tat basic proteins showed a greater degree of cellular entry compared to the transportan peptides. Also, it was deciphered that the mere cell-penetrating ability of CPPs is not indicative of their ability to traverse the BBB [71]. The blood–brain barrier is a tool for homeostasis and is selective to an extent wherein it is rendered almost impermeable [74]. As a result, certain small molecules/drugs and almost all large molecules cannot cross the BBB and hence cannot be used for therapeutic approaches. The first instance of the transport of a biologically active compound in the brain was shown by fusing the beta-galactosidase protein to the protein transduction domain (PTD) of the Tat-protein (Figure 5) [75]. Hence, experiments were conducted using conjugated drugs and the data obtained for CPP and nanomaterials showed that these conjugates could pave a path for treating CNS-associated disorders [76]. However, every advancement that has been introduced in drug delivery across the BBB has met multiple limitations and challenges owing to the complex design and the physiological impact of any disruptions that occur at the membrane. Notwithstanding these challenges, CPPs are coming up as potential tools for accomplishing such complicated drug deliveries.

Most of the earlier known CPPs are either covalent or non-covalent peptide-based delivery systems. Carrier peptides have many limitations: (i) lack of biocompatibility and bioavailability; (ii) may be toxic and antigenic; (iii) lack chemical fixation; (iv) may lose specificity to the target site; and (v) may get degraded by endosomes or proteasomes. In this context, MPG and Pep families of cell-penetrating small peptides have been successfully applied to the delivery of different cargoes (siRNA and peptides) both in vitro and in vivo, especially delivering therapeutics across the BBB. Listed are those CPPs that can act as a substitute for a covalent and non-covalent strategy for the delivery of drugs across the BBB.

#### 4.2.1. Lipoprotein-Enabled Novel Shuttle Peptides

Numerous novel shuttle peptides have been explored but efficient transport to the brain must be improvised, and researchers are still seeking the perfect approach to allow drugs to pass through the BBB. Lipoproteins seem to possess a significant ability as delivery systems to cross the BBB; for example, apolipoprotein B (ApoB), and apolipoprotein E (ApoE) equivalents were found to infiltrate the BBB [77,78]. Analogues of high-affinity lipid-associated peptides, namely, Ac-mE18A-NH2, Ac-hE18A-NH2, and Ac-hE(R)18A-NH2, tagged with hApoE, showed high internalisation compared to the control (Ac-R1018A-NH2), in which the receptor-binding domain contained only the positively charged arginine (R) [79]. Brain necrosis was significantly reduced in a mouse model with a therapeutic peptide (HAYED) that was an analogue of apolipoprotein E (K16APoE) tagged to 16 lysine (K16) residue linked to a low-density lipoprotein receptor-related protein (LDLR) [80].

#### 4.2.2. Naturally Derived CPPs

Amid the naturally occurring CPPs, virus-derived peptides have revolutionised targeted drug delivery across the BBB. A rabies virus glycoprotein (RVG-29) readily docks to the nicotinic acetylcholine (nAChR) receptor located on the endothelial cell lining and neuronal cells, thus facilitating its penetration across the BBB [81]. Overexpression of the α-synuclein (α-Syn) gene is the hallmark in Parkinson’s disease (PD) and the therapeutics pertain to the delivery of a shorter RVG linked to the negatively charged siRNA to suppress α-Syn. This version of RVG has a spacer of four additional glycine followed by positively charged arginine (R) at the end of the C-terminus (C2-9r (H2N-CDIFTNSRGKRAGGGGrrrrrrrrr, where r is D-arginine [82])). HIV-1-TAT peptidecan spontaneously internalise semiconductor nanowire (Si NW). TAT linked to surface SiNWs facilitates the internalisation of NW into mouse hippocampal neurons as well as into primary dorsal root ganglion (DRG) neurons [83]. Dengue virus type 2 capsid protein (*DEN2C*) can be used as a trans-BBB peptide vector as its translocation was shown to be receptor-independent while being steady with absorptive-mediated transport (AMT). One such peptide is PepH3, which shows tremendous potential for high brain penetration by crossing the BBB. This peptide is easily cleared from the brain via excretion; thus, it is a good candidate as a peptide shuttle to cargo in and out of the brain [84].

Other natural peptide-based shuttles are the venom-derived CPPs that have been demonstrated to traverse across the BBB and deliver drugs to the desired site. The monocyclic lactam-bridged peptidomimetic (MiniAp-4) analogue, derived from apamin (a neurological toxin from bee venom), was devised by minimising its intricacy, toxicity, immunogenicity, and protease resistance, while efficiently transporting drugs across the BBB into the brain parenchyma [85].

Nanoligand carrier (NLC), a brain-specific phage-derived peptide, is known to target cerebral endothelial cells via a transferrin receptor. Some phage peptides can recognise and bind their target, transit through the BBB, and reach neurons and microglial cells. NLC-β-secretase 1 (*BACE1*), another of the phage-displaying, self-assembled peptide siRNA complexes, displays effective *BACE1* suppression in the brain, without inflammation and/or toxicity. Therefore, to overcome limitations in specificity and efficacy, NLCs act as safe multifunctional CPPs or phage-display peptide nanocarriers [86]. A brain glioma cascade delivery system (AsTNP) was established by utilising an AS1411 aptamer and phage-displayed TGN peptide. The docetaxel-loaded AsTNP easily crossed the BBB and exhibited an anti-glioma effect with improved glioma survival [87]. Furthermore, two selected phage-display peptides, GLHTSATNLYLH and VAARTGEIYVPW, when co-cultured with primary rat endothelial cells and primary rat glial cells (astrocytes and microglia), crossed the BBB via active transport mechanisms [88].

#### 4.2.3. CPP-Mediated Nanocarriers

Peptidomimetic antibodies/ligands can be tailor-made by conjugating with nanocarriers that can identify transcytotic receptors on the membranous surface of the BBB for efficient delivery [89]. Nanoparticles (NPs) conjugated to drugs or diagnostics can encapsulate, adsorb, and get released at specific target sites/organs, including the brain. Biologically active polymeric NPs tagged with a TAT peptide (Tat-PEG-b-chol) can successfully deliver drugs across the BBB [90]. The polyamine (putrescine)-modified F(ab’) portion of an anti-amyloid antibody formulated with chitosan nanoparticles was also delivered to the brain [91]. Polymeric NPs (PMNPs) comprised of polysaccharides, proteins, amino acids, and polyesters are most extensively studied for brain drug delivery. PMNPs allow for transit across the BBB by either disrupting the tight junctions (TJs) and mucoadhesion in the brain capillaries or via transcytosis through brain endothelial cells [92]. Poly-ethylene glycol (PEG) liposomes are extensively used to conjugate with transferrin (Tf) and poly-L-arginine (cell-penetrating peptides) for delivering brain imaging drugs and DNA [93]. TfR-specific peptide B6 and endothelial growth factor receptor (EGFR) GE11 peptide can transport siRNA across the BBB [94]. 7-amino acid glycopeptide (g7) was used to deliver responsive angiopep-2-decorated poly(lactic-co-glycolic acid) (PLGA) hybrid NPs, while methoxypolyethylene glycol (MPEG) and methoxypoly (ethylene glycol)-b-polycaprolactone (PCL) NPs conjugated with angiopep-2 accumulated in the brain [95]. K16ApoE-decorated PLGA-NPs have shown a higher uptake into the brain and provided better MRI contrast for diagnostic purposes [96].

#### 4.2.4. CPP-Enabled Metallic Nanopeptides (NPs)

Metallic NPs are another form of nanocarriers that are extensively used to improve imaging as they can effectively cross the BBB. Also, glutathione (GSH)-conjugated iron NPs (GSHIONPs) forming IONPs@Asp-PTX-PEG-GSH are steady, non-toxic, and show improved MRI contrast for brain imaging [97]. In comparison to normal NPs, maleic anhydride-coated superparamagnetic iron oxide nanoparticles (Mal-SPIONs) showed improved dissemination to the thalamus, temporal lobe, and frontal cortex, [98]. Gold NPs conjugated to TAT (AuNPs-TAT) or glioma-specific peptide chlorotoxin (CTX) (Au PENPs) and showed improved cellular uptake in the brain [99]. Silicon NPs (pSiNPs) delivered siRNA across the BBB to treat brain gliomas with rabies virus-mimetic silica-coated gold nanorods [100].

#### 4.2.5. CPP-Enabled Exosomes

Exosomes are naturally produced by dendritic cells, monocytes, and macrophages with characteristic layers of lipids containing many adhesive proteins that help them interact well with the cellular membranes without getting entrapped within mononuclear phagocytes [101]. Thus, these have been explored to enhance the delivery of incorporated drugs to target cells, including the brain [102]. Exosomes derived from dendritic cells were used for combining neuron-specific RVG peptide tagged with lysosome-associated membrane protein 2b (*Lamp2b*) to carry siRNA into mouse brains. It was observed that serum levels for interleukin (IL)-6, tumour necrosis factor (TNF)-α, interferon gamma-induced protein (IP)-10, and interferon (IFN)-α serum substantially increased compared to those of siRNA-RVG-9R [103]. Dendritic cell-derived exosomes with interferon-γ were used to deliver miR-219, which increased myelination in rats’ brains [104]. The bioavailability of curcumin was increased by loading it onto exosomes, using it as a drug to treat brain lesions with cyclo-peptide (c(RGDyK)) [79,80], or as imaging material by conjugating it with a neuroleptin-1-targeted peptide [105]. Exosomes derived from bone marrow loaded with siRNA and RVG (targeting ligand) successfully decreased the α-Syn accumulation in the brain observed in patients with progressive Parkinson’s disease [106].

#### 4.2.6. CPP-Enabled Liposomes

In the last two decades, liposomes have been studied extensively as effective methods for drug delivery to the brain. Liposomal NPs usually get self-assembled within the phospholipid bilayer of the plasma membrane and can integrate into other biological membranes. The cationic liposome-siRNA-peptide (RVG-9r) containing cationic lipid octadecenolyoxy[ethyl-2-heptadecenyl-3 hydroxyethyl] imidazolinium chloride bound to the peptide moiety nAChRs penetrated the BBB to deliver siRNA into FVB mouse brains [107] with liposomes containing 1,2-dioleoyl-3-trimethylammonium-propane (DOTAP) or 1,2-distearoyl-sn-glycero-3-phosphoethanolamine (DSPE) complexed with siRNA and RVG peptides and target prions [108]. Cationic liposome-siRNA-peptide (RVG-9r) penetrated the BBB and reduced the effect of prion protein expression in the brain [109]. Analgesic peptides (kyotorphin or leu-enkephalin) self-assembled and encapsulated in a quaternary methyl ester derivative of methyl vernolate vesicles were successfully delivered to mouse brains [110]. Stable nucleic acid lipid particles (SNALPs) decorated with RVG-9r peptide liposomes crossed the BBB and delivered siRNA that eliminated mutant ataxin-3 (SCA3) in the brain of Machado–Joseph disease mouse models [111]. In certain tumour growths, liposomal receptor-related protein 1 (*LRP1*) was conjugated with GRN1005, a peptide drug that limited malignant growth [112].

#### 4.2.7. Angiopep-Conjugated Polyethyleneglycol-Adapted Polyamidoamine Dendrimer (PAMAM–PEG–Angiopep)

PAMAM–PEG–angiopep/DNA NPs are dendrimer nanoparticles that were combined with apolipoprotein A-I (ApoA-I) and NL4-peptide, shown to be efficient carriers across the BBB. PAMAM is a surface primary amino group and has shown clathrin- and caveolae-mediated endocytosis of the nanocarriers comprising angiopep peptides. Intravenous injection of dendrimer nanoparticles of PAMAM–PEG–angiopep loaded with pEGFP plasmid was given to mice. Compared to the control groups of PAMAM/DNA NPs, gene expression was observed in all four regions of the mouse brains for the PAMAM–PEG–angiopep/DNA NPs, although cationic dendrimers showed haemolytic activity and cell cytotoxicity [113]. A combination of short interfering RNA (siRNA) with polyamidoamine (PAMAM) dendrimers (D) was observed to achieve silencing activity. The silencing capacity of the complex depended on D generation (G4, G5, G6, and G7), ionic strength, and N/P ratio (nitrogen amines in D/phosphate in siRNA). This assay revealed that structurally stable complexes could be formed independent of the ionic strength with N/P ratios of 5 (for G4, G5) and 10 (for G6, G7) that could penetrate the brain with minimal cytotoxicity [114]. However, even low-generation lysine dendrons (G0 and G1) conjugated with ApoE-derived peptide traversed through the BBB without any significant cytotoxicity (as noticed in up to 400 μM concentrations) [115].

Peptide-based drug delivery systems across the BBB have pros and cons such as low alteration in the BBB integrity, specific targeting and reduced toxicity, and some concerns associated with serum stability. NPs, shuttle peptides, liposomes, exosomes, and dendrimers conjugated with CPPs have shown much-enhanced permeability across the BBB. Although advances have been achieved with CPPs to cross the BBB, it has been shown that in many cases, CPPs selectively cross the BBB, which does not qualify the peptides as having effective BBB-penetrating ability. The differential influx property exhibited by CPPs can be attributed to their cationic nature (presence or absence of arginine residues), physicochemical properties (secondary structure at the membrane interface), and biological properties (cellular uptake ability) [71]. The arena of targeting and crossing the BBB is a challenging yet promising field. In-depth understanding of drug properties (pharmacokinetics and pharmacodynamics) and BBB at the molecular level is paramount to design and develop a CNS drug. Notwithstanding the many advances in drug delivery systems, there is still an indispensable need for research into improved delivery systems with fewer limitations. Peptide-based delivery systems need further optimisation and high specificity for brain targeting.

### 4.3. Homing Peptides as Delivery Systems across the Blood–Brain Barrier (BBB)

**Figure 6:**
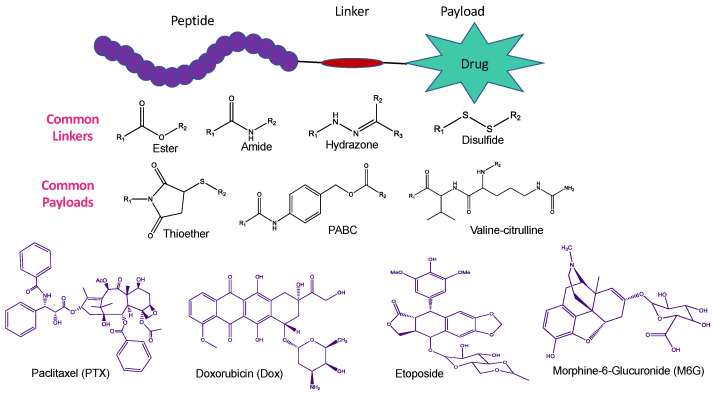
Illustration of several peptide–drug conjugates (PDCs), the chemical linkers, and the therapeutic payloads to which they are joined, and which can imitate an alternate antibody–drug conjugate (ADC).

Brain tumours and cancers are often fatal to patients because treatments such as chemotherapy suffer from poor bioavailability and reduced permeability across the BBB coupled with extensive intratumoral heterogeneity and a lack of selective tumour targeting. Peptide–drug conjugates (PDCs) are designed to link targeted peptides via a chemical linker to a therapeutic payload that can mimic an alternate antibody–drug conjugate (ADC) and expand the therapeutic potential of various drugs (Figure 6). In the context of BBB crossing and targeting CNS diseases, PDCs are designed to hijack the endogenous BBB influx transport mechanism and smuggle drugs into the brain parenchyma. Brain-permeable peptides or BBB shuttle peptides popularly known as brain-homing and brain-penetrating molecular transport vectors are a promising lot of molecules that can overcome the BBB and deliver drug molecules to the brain. Natural strategies like the phage, certain viruses, or natural neurotropic proteins can engage in receptor-mediated transcytosis for crossing the BBB. Thus, brain-homing peptides, linkers, and brain-permeable peptide–drug conjugates (PDCs) were shown to trick the brain by allowing the passage of molecules via the endogenous transcytosis mechanism [116].

Brain-homing peptide (BH) CNAFTPD is used to enhance the transfection efficacy of pDNA delivery across the BBB by forming biodegradable core–shell polyplexes with peptide–PEG–tris-acridine conjugates (pPAC) [117]. Similarly, a bacteria-based drug delivery system for glioblastoma (GBM) was employed as photothermal immunotherapy. Aptly called the ‘Trojan bacteria’, it was loaded with glucose polymer and photosensitive ICG silicon nanoparticles and shown to bypass the BBB, targeting and penetrating GBM tissues [118]. A brain-specific phage-derived peptide (nanoligand carrier, NLC) targets cerebral endothelial cells through the transferrin receptor and the receptor for advanced glycation end products. NLC-β-secretase 1 (*BACE1*) siRNA complexes are successfully delivered to neurons and microglial cells. Therefore, NLCs act as safe multifunctional nanocarriers with a wide receptor repertoire of the display peptide, which can effectively overcome the blood–brain barrier without toxicity and inflammation [119]. Recently, CNSRLHLRC, CENWWGDVC, and WRCVLREGPAGGCAWFNRHRL peptides were shown to mediate the selective localisation of phage to brain and kidney blood vessels. These peptide sequences identify selective endothelial markers to target drugs and genes in the brain and other selected tissues [120].

In glioblastoma (GBM), a debilitating brain tumour disease, a small, soluble peptide (BTP-7) covalently attaches to an insoluble anti-cancer drug, camptothecin (CPT), targeting the human GBM extracellular matrix (ECM) across the BBB [121]. Gliomas are other therapeutically problematic brain cancers with poor patient prognosis, and new drug delivery strategies are needed to achieve a more efficient chemotherapy-based approach against brain tumours. Using an in vitro phage display, fusion constructs with peptides and drugs forming Dox-SMCC-gHoPe2 have been studied where tumour-homing peptide gHo was identified as an efficient, in vivo-working vector [122]. A comprehensive summary of the cell-penetrating peptides and homing peptides used as brain drug delivery systems is provided in Table 1.

## 5. Cell-Penetrating Peptides and siRNA Delivery to the Central Nervous System

In the early 1990s, Napoli and Jorgensen reported posttranscriptional gene silencing in plants by RNA interference (RNAi). RNAi cleaves double-stranded or short hairpin RNA (shRNA) into functional, small interfering RNA (siRNA) with the aid of ‘dicer’, an endogenous mammalian protein complex that mediates RNAi. siRNAs are usually short (20–25 bp), exogenous, double-stranded RNA molecules that silence gene expression either by inhibiting transcription or by the degradation of sequence-specific target mRNA [128]. Since synthetic siRNAs can be intended against any target RNA, they have gained high therapeutic value. Indeed, siRNA-based therapeutics using small-molecule drugs can be positioned for the treatment of extensive neuronal diseases and cancers. siRNA therapeutics can also address the concerns usually posed by small-molecule drugs currently being used for targeted therapy. In vivo, targeted delivery of siRNA molecules remains a challenge due to its poor stability, reduced permeability across cellular membranes, poor endosomal escape, degradation by RNases, and rapid renal clearance. Therefore, to be effective delivery systems, siRNAs need a carrier for their protection from degradation [129]. Recently, many different approaches have been devised to deliver siRNAs into living cells by a broad variety of peptides. The simplicity of the synthesis, use, and versatility of CPPs have enabled siRNA delivery with promising strategies such as covalent conjugation, non-covalent complex formation, and CPP-decorated (functionalised) nano-complexes [130].

**Figure 7:**
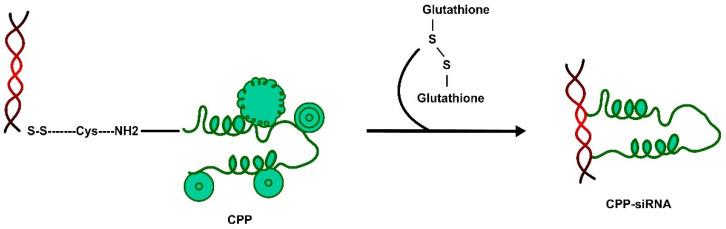
siRNA cargo covalently conjugated to the CPP is delivered via a cytosol-cleavable disulfide linkage that is separated in the reducing environment of the cytoplasm.

The first strategy is the **CPP covalently conjugated to the siRNA (CPP-siRNA)** delivery system. This method prevents separation among the CPP and its conjugate cargo both in vivo and/or in vitro. In this method, strong binding is attained between the CPP and its cargo via a cleavable linker such as the disulfide linkage, which leads to its lower molar ratio and low toxicity. This allows the components of the conjugate to be separated only in the reducing environment of the cytoplasm, e.g., by glutathione, thus avoiding its localisation in the nucleus (Figure 7) [131]. In contrast to the more cytotoxic liposome-based siRNA strategy, the penetratin-siRNA covalent approach was developed on neuron cells (in vitro) and the central nervous system (in vivo). However, there is still less clarity on the movement of siRNAs across the BBB and whether the internalisation and silencing effect is due to the covalently conjugated or the complexed species [132]. Apart from Tat and penetratin, siRNA conjugated to a low molecular weight protamine (LMWP) carrier via a cytosol-cleavable disulfide linkage using PEG as a spacer was developed, but this method was not successful [133].

The second strategy is the formation of **non-covalent complexes (CPP: siRNA),** which are designed for an optimal balance between the peptide and siRNA. The stability of the complex depends upon the structure of the CPP as it must avoid charge neutralisation, thus preserving an overall positive charge [134]. In this strategy, called CLIP-RNAi, the complexes can enter the cells via an endocytic pathway and later encourage endosomal escape via photo-stimulation, letting gene silencing [135]. Specific CPPs called homing peptides can bind to the vasculature of the tumour tissues and have been used to ‘aim’ them at a specific tissue of the central nervous system.

A short peptide derived from rabies virus glycoprotein (RVG) and conjugated with nona-arginine peptide (RVG-9R) could bind to the acetylcholine receptor expressed by neuronal cells, resulting in specific gene silencing within the brain [136]. A widespread study of siRNA transfection by means of nona-arginine (L and D) conjugated with diverse targeting ligands was evaluated for transfection and mRNA knockdown mechanism [136]. Another peptide myristic acid-conjugated transportan (TP) conjugated to transferrin receptor-targeting peptide (myr-TP-Tf) was successfully encapsulated, and siRNA was delivered to brain endothelial cells and glioma cells [137]. Notwithstanding these heartening outcomes, the use of siRNA and the advances in novel CPPs anticipated for targeted delivery have many challenges [138].

The third strategy is using **CPP-decorated multifunctional nano-complexes**, which use amalgamations of CPPs and other carriers for siRNA delivery. These multifunctional nano-complexes possess the characteristics of multiple compounds and have a better half-life in the bloodstream. Polyethylenimine (PEI) moiety tagged with a micelle-like nanoparticle (MNP) was replaced with nona-arginine (R9), forming lipid–peptide hybrid nanoparticles (hNPs) that could readily form complexes and transfer oligonucleotides. In addition, the hNPs modified with Tat 48–60 (T-hNP) were shown to improve cellular transfection. This formulation has been reported with a better gene-silencing effect in vivo as it readily accumulates in brain tumour tissue [139]. A stearylated octaarginine multifunctional envelope-type nano device (R8-MEND) was modified using a pH-sensitive fusogenic peptide GALA that facilitated endosomal escape when fused with small unilamellar vesicles (SUVs). R8:GALA-MENDSUV allowed RNA interference and downregulated the expression of the suppressor of the cytokine-signalling 1 (SOCS1) gene in primary mouse bone marrow-derived dendritic cells (BMDCs). Later, the R8-MEND system was equipped with a PEG–peptide–lipid ternary conjugate (PEG–peptide–DOPE conjugate (PPD) that acted as a PEG shield in the tumour tissue environment [140]. It was noted that in vivo, PPD-MEND clustered in tumours and exhibited silencing activity, with negligible hepatotoxicity and immune hyperactivity [141]. Another activatable CPP (DSPE-PEG2000-ACPP) liposome-based siRNA delivery system was developed that is made of an octaarginine peptide linked to a polyanionic ‘shielding domain’ of glutamate and histidine by means of an acid-labile linker (hydrazone). Within the mildly acidic pH in the tumour microenvironment, the linker is cleaved, and the histidine becomes protonated. This aids the interaction of octaarginine with the plasma membrane and lets the modified liposomes pass through the BBB and deliver the siRNA cargo [142]. To facilitate selective siRNA transfection both in vitro and in vivo, a photo-pH-responsive polypeptide (PPP) decorated with poly(lactic-co-glycolic acid) (PLGA) nanoparticles was developed. At the lower pH of the tumour environment, this system was exposed to infrared (NIR) that led to the cleaving of the photo-degradable group of CPPs and the freeing of the nanoparticle to traverse through the plasma membrane and deliver the siRNA in the cytoplasm [143].

## 6. siRNA-CPP Therapeutics of the Central Nervous System

### 6.1. siRNA Delivery by Virus

Additional challenges are posed to therapeutics targeted to specific cell types of CNS such as astrocytes, neurons, or glia cells to cross the blood–brain barrier (BBB). Hence, early attempts to deliver RNAi in the CNS were performed by intracranial injections of lentiviral vectors encoding shRNA, but the method showed diminished efficacy. Lentiviral vectors crossed the BBB poorly. The inadequate neuro-invasion led to decreased and localised hRNA expression only around the injection site. This method also lacked temporal control and caused limited knockdown of protein expression. Another major concern regarding lentiviral vector technology and construction is its capacity to turn brain cells cancerous [144,145]. Another small, modified peptide that reduces siRNA off-target effects is obtained from the rabies virus glycoprotein (RVG-9r). Additionally, nicotinic acetylcholine receptors in the CNS are targeted by cationic liposomes with siRNA peptide complexes (LSPCs) and a targeting peptide (RVG-9r). Some modified siRNA-peptide complexes are encapsulated in either cationic or anionic liposomes with RVG-9r and bonded to lipid PEG either electrostatically or covalently; these are called peptide-addressed liposome-encapsulated therapeutic siRNA (PALETS) [146]. It was proven that LSPCs and PALETS reduced the surface cellular prion protein (PrPC) up to 70% in neuronal cells, while PALETS downregulated the total number of PrPC-expressing cells.

### 6.2. Non-Viral Route of siRNA Delivery

Cationic small-cell-penetrating peptides capable of crossing plasma membranes were the alternate nonviral strategy used for delivering siRNA molecules to the CNS [147,148]. The thiol linkages allow the siRNA–peptide complexes to easily dissociate in the cytoplasm by reduction of the disulfide bond. To overcome this, liposomal siRNA delivery vehicles conjugated to peptides were designed for transport to the CNS by means of the thin-film hydration method. The cationic or anionic liposomes saved the siRNAs from nucleases and proteases, whereas the combination with a peptide helped them bind to nicotinic acetylcholine receptors (nAchRs) on the brain cell surface [136]. Notwithstanding the therapeutic possibilities, this method remains plagued by important concerns regarding drug delivery to the brain such as cell specificity and transport of its cargo across the BBB. Most of these naked complexes become significantly degraded in the blood during transport as there was little or no detection of siRNA in the treated mouse brains. Moreover, there is also the problem of these complexes getting immune cleared or degraded by serum nuclease and protease.

### 6.3. Liposome–siRNA–Peptide Complexes (LSPCs)

Alternatively, more direct routes to the CNS should be explored and, in this regard, liposomes are extensively used as delivery vehicles for siRNA. Improved delivery of the liposome-encapsulated siRNA was observed with minimal siRNA degradation within the blood [149]. Cationic liposomes are the most common choice as they interact both with the anionic phosphate head groups of cell membranes and the negatively charged phosphate backbone of RNA [150,151]. Although less immunogenic, anionic liposomes are used less frequently because they repel the negative charge of the siRNA backbone and cannot penetrate the plasma membrane [152]. Liposome–siRNA complexes covalently bound to peptides have an affinity for receptors on specific cell types and thus can avoid off-target binding [153]. The mononuclear phagocyte system of the immune system has the tendency to recognise serum proteins and engulfing liposomes. Thus, PEGylated liposomes can avoid immune clearance and increase bioavailability and circulation duration in the blood [154]. However, bare liposomes are neither transported across the BBB nor provide cell-specific delivery. Surprisingly, PEGylated liposomes are known to reduce the serum degradation of siRNA to target sites in the brain, but coupling with a monoclonal antibody against glial fibrillary acidic protein (GFAP) failed to deliver itself across the astrocytes in mouse brains [155]. Thus, an effective and efficient delivery system must (1) gain access to the brain by crossing the BBB, (2) provide a molecular address for delivery to neuronal cells, and (3) protect from serum degradation.

### 6.4. Intranasal Delivery of siRNA

Systemic transit through the vasculature of the CNS may lead therapeutic drugs to encounter immune cells, causing hypersensitivity or clearance from the system. An alternate method of administering drugs or stem cells into the nasal cavity can shorten the route across the BBB [156,157,158]. Neurodegenerative diseases like Alzheimer’s and Parkinson’s were shown to be treated by delivering mesenchymal stem cells intranasally [159,160]. Therapeutic targeting with a more direct route to the CNS combined with specific delivery to brain cells has been observed in other brain pathologies. Brain tumours and perinatal and ischaemic brain damage can also be treated using intranasally administrated mesenchymal stem cells [161,162,163].

### 6.5. siRNA-Loaded Exosomes

Viral vectors or liposomes fail to deliver specifically and safely into the brain as their approaches are invasive and hindered by the immune system. Liposomes lack a long-term treatment method as they tend to elicit immune responses followed by subsequent clearance, while the use of a viral vector requires stereotaxic surgery, and the delivery is restricted to specific brain regions. These disadvantages can be overcome by the intravenous (iv) injection of modified exosomes for nucleic acid delivery into the brain. Exosomes are nanosized extracellular vesicles (30–100 nm in diameter) formed by an endocytic pathway [164]. Exosomes represent a promising drug delivery system and act as natural carriers of mRNA, microRNA, and proteins between cells [165]. Izco and co-workers developed a modified exosome that specifically targets the brain by delivering genetic material into the brain via intravenous injection. siRNAs-RVG-exosomes have been delivered for an effective knockdown of expression for both the Aβ and tau peptides of Alzheimer’s disease and the alpha-synuclein of Parkinson’s disease as a long-term treatment for these neurodegenerative diseases [166]. However, it was also noted that a defective endolysosomal system may interfere with the delivery of siRNAs. Thus, strategies must be devised to not only inhibit exosome secretion but also modify the content of exosomes by decreasing the exosomal cargo of pathological proteins, neuroinflammatory factors, or altered miRNAs. Increasing the cargo of trophic factors in glial-derived exosomes could also create new therapeutic strategies to halt the progression of neurodegenerative diseases [167].

Although the abovementioned siRNA delivery strategies have proven to be successful, some of the basic guidelines will become clearer after elaborating on the in vivo studies and clinical trials (Table 2). In the case of complexation-based systems, PEGylation or similar surface modification is necessary for longevity in circulation to shield the positive charge. This prevents nonspecific interaction and reduces toxicity. However, after delivering the siRNA to the target site, it must ensure endo/lysosomal escape following internalisation by the target cells. Despite the cost and regulatory hurdles associated with the targeting strategy, the siRNA-CPP delivery system is a big incentive, and the benefits are irrefutable [168].

## 7. Conclusions and Perspectives

Diseases of the central nervous system (CNS) are the most difficult to treat, mainly because of the obstacle of the blood–brain barrier (BBB). A vast majority of drugs fail to reach the brain because of their inability to cross the BBB. Undoubtedly, the most promising studies are those that unravel strategies to deliver CNS-active drugs and peptides targeted at the BBB. Small peptides as nanoparticles or nanocarriers can be conjugated to drugs to either form a steady link or act as pro-drugs. The last decade witnessed a plethora of uses of both small molecules and proteins in diverse therapeutic areas such as diagnostics, brain cancers, and neurodegenerative disorders. Peptides can be generally classified as ‘receptor-targeted’ (e.g., angiopep2, CDX, and iRGD), recognising membrane proteins expressed by the BBB microvessels; ‘cell-penetrating peptides’ (e.g., TAT47−57, SynB1/3, and Penetratin), undergoing transcytosis through unspecific mechanisms; or ‘homing peptides’ (e.g., glioma-homing peptide (gHo) NHQQQNPHQPPM; brain-homing peptides CNAFTPDY, CLEVSRKNC, and CLSSRLDAC), used for DNA delivery. RNA interference (RNAi) conjugated to CPPs can become a new tool to target defective genes in the brain by inducing gene silencing and has enormous clinical potential for the treatment of various neurodegenerative disorders. Most RNAi trials use small interfering RNAs (siRNAs) but, despite the enormous potential of RNAi, only 24 clinical trials have applied siRNA-CPP-mediated therapy to date. Notwithstanding their great therapeutic potential, the successful implementation of siRNAs in vivo is hampered by the low bioavailability of these hydrophilic compounds and their inability to cross the BBB [169]. We hope that future directions of peptide therapy will offer a completely different approach to treating these progressive neurodegenerative illnesses and change the lives of those with these debilitating conditions.

In theory, both CPPs and homing peptides can be exploited to transform a large range of pharmaceuticals from paper to practice, but there exists a hindrance when it comes to replicating the in vitro results within physiological systems. Undeniably, despite having a generally good safety profile, some peptide conjugates may display toxicological features, causing antigenicity, cardiovascular alterations, or hemolysis. Furthermore, the life span of these peptides largely depends on their dodging capability in the presence of peptidases, endosomal/lysosomal degradation, and endosomal entrapment, making them unavailable to the target site [69].

Despite numerous data pertaining to the basics and applications of CPPs, the efficacy of such conjugations is still debatable, primarily when it comes to the blood–brain barrier. Endogenous analysis of CPP-mediated drug targeting in cell cultures does not necessarily indicate their behaviour at the organismal level. As discussed in the text, mere penetration inside cells does not mean that a given CPP can traverse the blood–brain barrier. Such limitations necessitate further in-vivo analysis of multiple CPPs as well as additional penetrating peptides and expand the existing database. The study of CPPs should also consider the differential expression of transporter proteins, primarily solute carriers on cell surfaces since the expression levels of these transmembrane proteins are strongly associated with various normal and anomalous conditions. Certain biologically active chemical compounds might also be studied for their cell-penetration and transcellular traversing activities so that the additional conjugation step can be eliminated.

Even though modern-day medicine has tapped the potential of many physiological loopholes to the advantage of humankind, a broad area remains unexplored. A significant proportion of our physiology and metabolism is influenced by the dark matter in our system. Despite null results, unknown interactions can also lead to negative results, which are commonly referred to as side effects. For most pharmaceuticals, these side effects exceed what has been documented or studied. One way to minimise these undocumented results is to take the dark matter into consideration. This is possible owing to the CPP/homing peptide specificity. However, despite this specificity, drugs can frequently target more than one macromolecule in an organism. Additionally, drug-bound CPPs can exhibit altered behaviours that further interfere with their normal roles in biological systems, specifically, brain activity. This can be negated by using pre-conjugated drug–CPP or drug–homing peptide combinations to minimise unwanted interactions within physiological systems. The careful optimisation of this and additional techniques can aid in moving many potential pharmaceuticals from texts to tables. An underestimated yet large number of efficient drugs fail to reach the counters owing to the lack of a delivery mechanism. Potent drugs (biomolecules and nanoparticles) that can be used to cure fatal disorders of the CNS, such as Parkinson’s and Alzheimer’s disease, or treat various brain tumours, do not exhibit any significant results if not coupled with an adequate transport and translocation system. Thus, a detailed analysis of both the available and the potential drug delivery systems that can effectively cross the blood–brain barrier has emerged as a critical requirement in the world of pharma- and nutraceuticals.

## Figures and Tables

**Table 1:** List of probable CPP/homing peptides and their sequence, source, and formulations used as a target molecule against the blood–brain barrier.

Name of the Peptide	Sequence of the Peptide	Peptide Source	Formulations/Carriers	Ref. No.
ApoE	LRKLRKRLL	Apolipoprotein E	Shuttle synthetic peptides	[77]
ApoB	SSVIDALQYKLEGTTRLTRKRGLKLATALSLSNKFVEGS	Apolipoprotein B	Shuttle synthetic peptides	[78]
hApoE	LRKLRKRLLR	Human apolipoprotein E (hApoE)	Shuttle synthetic peptides	[79]
RVG-29	YTIWMPENPRPGTPCDIFTNSRGKRASNG	Rabies virus glycoprotein	Shuttle natural peptide	[81]
TAT	GGGGYGRKKRRQRRR	Human immunodeficiency virus 1	Shuttle natural peptide	[83]
PepH3	AGILKRW	Dengue virus type 2 capsid protein (*DEN2C*)	Shuttle natural peptide	[84]
Apamin	H-CNCKAPETALCARRCQQH-NH2	Venom neurotoxin	Shuttle natural peptide	[85]
MiniAp-4	H-DapKAPETALD-NH2	Venom neurotoxin	Shuttle natural peptide	[85]
THRre	PWVPSWMPPRHT	Phage display	Shuttle natural peptide	[86]
TGN	TGNYKALHPHNG	Phage display	Shuttle natural peptide	[87]
THR	THRPPMWSPVWP	Phage display	Shuttle natural peptide	[123]
THRre_2f	(PWVPSWMPPRHT)2KKGK(CF)G	Phage display	Shuttle natural peptide	[124]
K16APoE	HAYED	Apolipoprotein E (LDLR)	Shuttle natural peptide	[125]
TAT peptide	Tat-PEG-b-chol	Nanoparticles	NPs (PMNPs)	[90]
Polyamine (putrescine)	F(ab’) anti-amyloid antibody	Nanoparticles	Polymeric NPs (PMNPs)	[91]
TfR-peptide	TfR poly-L-arginine	Poly-ethylene glycol liposomes	Polymeric NPs (PMNPs)	[93]
GE11 peptide	TfR-endothelial factor receptor (EGFR)	siRNA/TMC–PEG-RV	Polymeric NPs (PMNPs)	[94]
Angiopep-2	TFFYGGSRGKRNNFKTEEY	Neurotropic endogenous protein	Polymeric NPs (PMNPs)	[95]
K16APoE	HAYED	PLGA-NPs	Polymeric NPs (PMNPs)	[96]
g7	GFtGPLS (O-β-d-glucose) CONH2	Enkephalin analogues/opioid	Polymeric NPs (PMNPs)	[119,126]
Mal-SPIONs	[C_2_H_2_(CO)_2_O]Fe₂O₃	Superparamagnetic iron oxide nanoparticles	Metallic NPs	[88]
GSH-peptide	IONPs@Asp-PTX-PEG-GSH	Glutathione nanoparticles (GSHIONPs)	Metallic NPs	[97]
Silicon NPs	pSiNPs	Rabies virus-mimetic silica-coated gold nanorods	Metallic NPs	[100]
cyclo-peptide	c(RGDy)K	Macrophages/monocytes	Exosomes	[80]
neuron-specific RVG peptide	siRNA-RVG-9R	Dendritic cells	Exosomes	[103]
miR-219		Dendritic cells	Exosomes	[104]
siRNA3 RVG		Bone marrow	Exosomes	[106]
siRNA-peptide	octadecenolyoxy[ethyl-2-heptadecenyl-3 hydroxyethyl] imidazolinium chloride	Bone marrow	Exosomes	[107]
neuroleptin-1-targeted peptide	RGERPRR	Macrophages/monocytes	Exosomes	[127]
siRNA-RVG peptide	1,2-dioleoyl-3-trimethylammonium-propane (DOTAP)	Cationic liposomes	Liposomes	[108]
siRNA-RVG peptide	1,2-distearoyl-sn-glycero-3-phosphoethanolamine (DSPE)	Cationic liposomes	Liposomes	[108]
siRNA-peptide (RVG-9r)	RVG-29-PEG-PLGA/DTX	Cationic liposomes	Liposomes	[109]
kyotorphin or leu-enkephalin	methyl ester-methyl vernolate	Self-assembled liposomes	Liposomes	[110]
siRNA-RVG peptide	Stable nucleic acid lipid particles [SNALPs]	Self-assembled liposomes	Liposomes	[111]
*LRP1*	ANG-PEG– poly(ε-caprolactone)	Self-assembled liposomes	Liposomes	[102,112]
Angiopep peptide	TFFYGGSRGKRNNFKTEEYC	PAMAM–PEG–Angiopep/DNA	Dendrimer nanoparticles	[113]
ApoE derived peptide	LRKLRKRLLR	Lysine dendrons	Dendrimer nanoparticles	[115]
pPAC	CNAFTPD	Peptide-PEG-tris-acridine conjugates (pPAC)	Brain-homing peptide (BH)	[117]
phage-derived peptide	NLC-β-secretase 1 (*BACE1*) siRNA	Photosensitive ICG silicon-nanoparticles	Brain-homing peptide (BH)	[119]
phage-derived peptide	CNSRLHLRC, CENWWGDVC, WRCVLREGPAGGCAWFNRHRL	Nanoparticles	Brain-homing peptide (BH)	[120]
BTP-7	BTP-7-Camptothecin (CPT)	Patient-derived GBM stem cells	Brain-homing peptide (BH)	[121]
gHoPe2	NHQQQNPHQPPM	Phage-derived	Glioma-homing peptide (gHo)	[122]

**Table 2:** A list of various formulations and presumptive concerns for the use of siRNA–peptide conjugates.

S. No.	siRNA-CPP Therapeutics	Route of Delivery	Formulations	Consequences/Concerns	Refs.
1.	Virus-delivered siRNAs				
	Lentivirus vector	Intracranial injections of hRNA to CNS	Vesicular stomatitis virus glycoprotein envelope (VSV-G)	Can turn brain cells cancerous	[144]
	b. RVG-9r	Intravascular injection targeted to neuronal PrPC	siRNA encapsulated in either cationic or anionic liposomes	Decreased levels of cellular prion protein (PrPC)	[146]
2.	Non-viral delivery of siRNAs	Intravenous administration of cholesterol-conjugated siRNA lipoplexes	Cationic, anionic, or neutral, or a mixture, liposomes	Significant degradation during blood transport. Degradation by serum nuclease and protease. Immune clearance.	[148]
3.	Liposome-siRNA-peptide complexes (LSPCs)	In vitro RNA transfection with DOTMA-containing liposomes (lipofectin)	Cationic liposome	Immune clearance. Poor transport capability.	[149]
		In vitro transfection with anionic lipoplexes (DOPG:DOPE)	Anionic liposome	Repel the negative charge of the siRNA backbone. Poor penetration through plasma membrane.	[152]
		Intravenous injection	PEGylated liposomes	Good bioavailability. No immune clearance.	[154]
		Intravenous injection	PEGylated liposomes plus monoclonal antibody	Poor transport across the astrocytes in mouse brains.	[155]
4.	Intranasal delivery of siRNA	Direct administration of drugs or stem cells into the nasal cavity	Human bone marrow-derived mesenchymal stem cells (MSC)	Shorter route to CNS.	[159,160,161]
5.	siRNA-loaded exosomes	Intravenous injection	Exosomes	Increased cargo interferes with endosomal system.	[164]

## Data Availability

Not applicable.

## References

[1] Bhowmik A., Khan R., Ghosh M.K. (2015). Blood brain barrier: A challenge for effectual therapy of brain tumours. BioMed. Res. Int..

[2] Lu Y., Zhang E., Yang J., Cao Z. (2018). Strategies to improve micelle stability for drug delivery. Nano. Res..

[3] Morshedi Rad D., Alsadat Rad M., Razavi Bazaz S., Kashaninejad N., Jin D., Ebrahimi Warkiani M. (2021). A comprehensive review on intracellular delivery. Adv. Mater..

[4] Stewart M.P., Sharei A., Ding X., Sahay G., Langer R., Jensen K.F. (2016). In vitro and ex vivo strategies for intracellular delivery. Nature.

[5] Berillo D., Yeskendir A., Zharkinbekov Z., Raziyeva K., Saparov A. (2021). Peptide-based drug delivery systems. Medicina.

[6] Gayraud F., Klugman M., Neundorf I. (2021). Recent Advances and Trends in Chemical CPP–Drug Conjugation Techniques. Molecules.

[7] Josephson L., Tung C.H., Moore A., Weissleder R. (1999). High-efficiency intracellular magnetic labeling with novel superparamagnetic-Tat peptide conjugates. Bioconjug. Chem..

[8] Blasi P., Giovagnoli S., Schoubben A., Ricci M., Rossi C. (2007). Solid lipid nanoparticles for targeted brain drug delivery. Adv. Drug Deliv. Rev..

[9] Malhotra M., Prakash S. (2011). Targeted drug delivery across blood-brain-barrier using cell penetrating peptides tagged nanoparticles. Curr. Nanosci..

[10] Goyal R. (2020). Peptide-Based Molecular Constructs for Cellular Targeting and Small Molecule Delivery. Ph.D. Thesis.

[11] Heitz F., Morris M.C., Divita G. (2009). Twenty years of cell-penetrating peptides: From molecular mechanisms to therapeutics. Br. J. Pharmacol..

[12] Frankel A.D., Pabo C.O. (1988). Cellular uptake of the TAT protein from human lmmunodeficiency virus. Cell.

[13] Nagahara H., Vocero-Akbani A.M., Snyder E.L., Ho A., Latham D.G., Lissy N.A., Becker-Hapak M., Ezhevsky S.A., Dowdy S.F. (1998). Transduction of full-length TAT fusion proteins into mammalian cells: TAT-p27Kip1 induces cell migration. Nat. Med..

[14] Morris M.C., Deshayes S., Heitz F., Divita G. (2008). Cell-penetrating peptides: From molecular mechanisms to therapeutics. Biol. Cell.

[15] Ndeboko B., Ramamurthy N., Lemamy G.J., Jamard C., Nielsen P.E., Cova L. (2017). Role of cell-penetrating peptides in intracellular delivery of peptide nucleic acids targeting hepadnaviral replication. Mol. Ther.-Nucleic Acids.

[16] Zhang D., Wang J., Xu D. (2016). Cell-penetrating peptides as noninvasive transmembrane vectors for the development of novel multifunctional drug-delivery systems. J. Control. Release.

[17] Komin A., Russell L., Hristova K., Searson P. (2017). Peptide-based strategies for enhanced cell uptake, transcellular transport, and circulation: Mechanisms and challenges. Adv. Drug Deliv. Rev..

[18] Arukuusk P., Pärnaste L., Margus H., Eriksson N.J., Vasconcelos L., Padari K.R., Pooga M., Langel U.L. (2013). Differential endosomal pathways for radically modified peptide vectors. Bioconjugate Chem..

[19] Ruseska I., Zimmer A. (2020). Internalization mechanisms of cell-penetrating peptides. Beilstein J. Nanotechnol..

[20] Hu G., Zheng W., Li A., Mu Y., Shi M., Li T., Zou H., Shao H., Qin A., Ye J. (2018). A novel CAV derived cell-penetrating peptide efficiently delivers exogenous molecules through caveolae-mediated endocytosis. Vet. Res..

[21] Haucke V., Kozlov M.M. (2018). Membrane remodeling in clathrin-mediated endocytosis. J. Cell Sci..

[22] Kaksonen M., Roux A. (2018). Mechanisms of clathrin-mediated endocytosis. Nat. Rev. Mol. Cell Biol..

[23] Futaki S., Nakase I. (2017). Cell-surface interactions on arginine-rich cell-penetrating peptides allow for multiplex modes of internalization. Acc. Chem. Res..

[24] Guidotti G., Brambilla L., Rossi D. (2017). Cell-penetrating peptides: From basic research to clinics. Trends Pharmacol. Sci..

[25] Böhmová E., Machová D., Pechar M., Pola R., Venclíková K., Janoušková O., Etrych T. (2018). Cell-penetrating peptides: A useful tool for the delivery of various cargoes into cells. Physiol. Res..

[26] Erazo-Oliveras A., Muthukrishnan N., Baker R., Wang T.-Y., Pellois J.-P. (2012). Improving the endosomal escape of cell-penetrating peptides and their cargos: Strategies and challenges. Pharmaceuticals.

[27] Smith S.A., Selby L.I., Johnston A.P.R., Such G.K. (2019). The Endosomal Escape of Nanoparticles: Toward More Efficient Cellular Delivery. Bioconjugate Chem..

[28] Varkouhi A.K., Scholte M., Storm G., Haisma H.J. (2011). Endosomal escape pathways for delivery of biologicals. J. Control. Release.

[29] Martens T.F., Remaut K., Demeester J., De Smedt S.C., Braeckmans K. (2014). Intracellular delivery of nanomaterials: How to catch endosomal escape in the act. Nano Today.

[30] Meyer M., Philipp A., Oskuee R., Schmidt C., Wagner E. (2008). Breathing life into polycations: Functionalization with pH-responsive endosomolytic peptides and polyethylene glycol enables siRNA delivery. J. Am. Chem. Soc..

[31] Kobayashi S., Nakase I., Kawabata N., Yu H.-H., Pujals S., Imanishi M., Giralt E., Futaki S. (2009). Cytosolic targeting of macromolecules using a pH-dependent fusogenic peptide in combination with cationic liposomes. Bioconjugate Chem..

[32] Chen R., Khormaee S., Eccleston M.E., Slater N.K. (2009). The role of hydrophobic amino acid grafts in the enhancement of membrane-disruptive activity of pH-responsive pseudo-peptides. Biomaterials.

[33] Lonn P., Kacsinta A., Cui X., Hamil A., Kaulich M., Gogoi K., Dowdy S. (2016). Enhancing endosomal escape for intracellular delivery of macromolecular biologic therapeutics. Sci. Rep..

[34] Han K., Chen S., Chen W.-H., Lei Q., Liu Y., Zhuo R.-X., Zhang X.-Z. (2013). Synergistic gene and drug tumour therapy using a chimeric peptide. Biomaterials.

[35] Lo S.L., Wang S. (2008). An endosomolytic Tat peptide produced by incorporation of histidine and cysteine residues as a nonviral vector for DNA transfection. Biomaterials.

[36] Qian Z., LaRochelle J.R., Jiang B., Lian W., Hard R.L., Selner N.G., Luechapanichkul R., Barrios A.M., Pei D. (2014). Early endosomal escape of a cyclic cell-penetrating peptide allows effective cytosolic cargo delivery. Biochemistry.

[37] Erazo-Oliveras A., Najjar K., Truong D., Wang T.Y., Brock D.J., Prater A.R., Pellois J.P. (2016). The Late Endosome and Its Lipid BMP Act as Gateways for Efficient Cytosolic Access of the Delivery Agent dfTAT and Its Macromolecular Cargos. Cell Chem. Biol..

[38] Erazo-Oliveras A., Najjar K., Dayani L., Wang T.Y., Johnson G.A., Pellois J.P. (2014). Protein delivery into live cells by incubation with an endosomolytic agent. Nat. Methods.

[39] Qian Z., Martyna A., Hard R.L., Wang J., Appiah-Kubi G., Coss C., Phelps M.A., Rossman J.S., Pei D. (2016). Discovery and Mechanism of Highly Efficient Cyclic Cell-Penetrating Peptides. Biochemistry.

[40] Appelbaum J.S., LaRochelle J.R., Smith B.A., Balkin D.M., Holub J.M., Schepartz A. (2012). Arginine topology controls escape of minimally cationic proteins from early endosomes to the cytoplasm. Chem. Biol..

[41] Bus T., Traeger A., Schubert U.S. (2018). The great escape: How cationic polyplexes overcome the endosomal barrier. J. Mater. Chem. B.

[42] Pack D.W., Putnam D., Langer R. (2000). Design of imidazole-containing endosomolytic biopolymers for gene delivery. Biotechnol. Bioeng..

[43] Sonawane N.D., Szoka F.C., Verkman A.S. (2003). Chloride accumulation and swelling in endosomes enhances DNA transfer by polyamine-DNA polyplexes. J. Biol. Chem..

[44] Benjaminsen R.V., Mattebjerg M.A., Henriksen J.R., Moghimi S.M., Andresen T.L. (2013). The possible “proton sponge“ effect of polyethylenimine (PEI) does not include change in lysosomal pH. Mol. Ther..

[45] Vermeulen L.M.P., De Smedt S.C., Remaut K., Braeckmans K. (2018). The proton sponge hypothesis: Fable or fact?. Eur. J. Pharm. Biopharm..

[46] Tweten R.K. (2005). Cholesterol-dependent cytolysins, a family of versatile pore-forming toxins. Infect. Immun..

[47] Gruenberg J., van der Goot F.G. (2006). Mechanisms of pathogen entry through the endosomal compartments. Nat. Rev. Mol. Cell Biol..

[48] Herce H.D., Garcia A.E., Litt J., Kane R.S., Martin P., Enrique N., Rebolledo A., Milesi V. (2009). Arginine-rich peptides destabilize the plasma membrane, consistent with a pore formation translocation mechanism of cell-penetrating peptides. Biophys. J..

[49] Nadal-Bufí F., Henriques S.T. (2020). How to overcome endosomal entrapment of cell-penetrating peptides to release the therapeutic potential of peptides?. Pept. Sci..

[50] Mandal D., Nasrolahi Shirazi A., Parang K. (2011). Cell-penetrating homochiral cyclic peptides as nuclear-targeting molecular transporters. Angew. Chem. Int. Ed. Engl..

[51] Lee Y.J., Johnson G., Peltier G.C., Pellois J.P. (2011). A HA2-Fusion tag limits the endosomal release of its protein cargo despite causing endosomal lysis. Biochim. Biophys. Acta.

[52] Song J., Lu C., Leszek J., Zhang J. (2021). Design and Development of Nanomaterial-Based Drug Carriers to Overcome the Blood–Brain Barrier by Using Different Transport Mechanisms. Int. J. Mol. Sci..

[53] Lucana M.C., Arruga Y., Petrachi E., Roig A., Lucchi R., Oller-Salvia B. (2021). Protease-Resistant Peptides for Targeting and Intracellular Delivery of Therapeutics. Pharmaceutics.

[54] Varner J.A., Cheresh D.A. (1996). Integrins and cancer. Curr. Opin. Cell Biol..

[55] Koivunen E., Wang B., Ruoslahti E. (1995). Phage libraries displaying cyclic peptides with different ring sizes: Ligand specificities of the RGD-directed integrins. Biotechnology.

[56] Corti A., Curnis F. (2011). Tumour vasculature targeting through NGR peptide-based drug delivery systems. Curr. Pharm. Biotechnol..

[57] Kondo E., Iioka H., Saito K. (2021). Tumour-homing peptide and its utility for advanced cancer medicine. Cancer Sci..

[58] Kondo E., Saito K., Tashiro Y., Kamide K., Uno S., Furuya T., Mashita M., Nakajima K., Tsumuraya T., Kobayashi N. (2012). Tumour lineage-homing cell-penetrating peptides as anticancer molecular delivery systems. Nat. Commun..

[59] Pardridge W.M. (2005). The blood-brain barrier: Bottleneck in brain drug development. NeuroRx.

[60] Treat L.H., McDannold N., Zhang Y., Vykhodtseva N., Hynynen K. (2012). Improved anti-tumour effect of liposomal doxorubicin after targeted blood-brain barrier disruption by MRI-guided focused ultrasound in rat glioma. Ultrasound. Med. Biol..

[61] Rip J., Schenk G.J., de Boer A.G. (2009). Differential receptor-mediated drug targeting to the diseased brain. Expert. Opin. Drug. Deliv..

[62] Gururangan S., Friedman H.S. (2002). Innovations in design and delivery of chemotherapy for brain tumours. Neuroimaging Clin. N. Am..

[63] Saraiva C., Praça C., Ferreira R., Santos T., Ferreira L., Bernardino L. (2016). Nanoparticle-mediated brain drug delivery: Overcoming blood-brain barrier to treat neurodegenerative diseases. J. Control. Release.

[64] Sweeney M.D., Ayyadurai S., Zlokovic B.V. (2016). Pericytes of the neurovascular unit: Key functions and signaling pathways. Nat. Neurosci..

[65] Abbott N.J., Romero I.A. (1996). Transporting therapeutics across the blood-brain barrier. Mol. Med. Today.

[66] Abbott N.J., Rönnbäck L., Hansson E. (2006). Astrocyte-endothelial interactions at the blood-brain barrier. Nat. Rev. Neurosci..

[67] Pajouhesh H., Lenz G.R. (2005). Medicinal chemical properties of successful central nervous system drugs. NeuroRx.

[68] Lipinski C.A., Lombardo F., Dominy B.W., Feeney P.J. (2001). Experimental and computational approaches to estimate solubility and permeability in drug discovery and development settings. Adv. Drug Deliv. Rev..

[69] Parrasia S., Szabò I., Zoratti M., Biasutto L. (2022). Peptides as Pharmacological Carriers to the Brain: Promises, Shortcomings and Challenges. Mol. Pharm..

[70] Reissmann S. (2021). State of art: Cell penetration and cell-penetrating peptides and proteins. Health Educ. Public Health.

[71] Stalmans S., Bracke N., Wynendaele E., Gevaert B., Peremans K., Burvenich C., Polis I., De Spiegeleer B. (2015). Cell-Penetrating Peptides Selectively Cross the Blood-Brain Barrier In Vivo. PLoS ONE.

[72] Lindgren M.E., Hällbrink M.M., Elmquist A.M., Langel U. (2004). Passage of cell-penetrating peptides across a human epithelial cell layer in vitro. Biochem. J..

[73] Violini S., Sharma V., Prior J.L., Dyszlewski M., Piwnica-Worms D. (2002). Evidence for a plasma membrane-mediated permeability barrier to Tat basic domain in well-differentiated epithelial cells: Lack of correlation with heparan sulfate. Biochemistry.

[74] Chen Y., Liu L. (2012). Modern methods for delivery of drugs across the blood-brain barrier. Adv. Drug Deliv. Rev..

[75] Schwarze S.R., Ho A., Vocero-Akbani A., Dowdy S.F. (1999). In vivo protein transduction: Delivery of a biologically active protein into the mouse. Science.

[76] Zhang Y., Guo P., Ma Z., Lu P., Kebebe D., Liu Z. (2021). Combination of cell-penetrating peptides with nanomaterials for the potential therapeutics of central nervous system disorders: A review. J. Nanobiotechnol..

[77] Datta G., Chaddha M., Garber D.W., Chung B.H., Tytler E.M., Dashti N., Bradley W.A., Gianturco S.H., Anantharamaiah G.M. (2000). The receptor binding domain of apolipoprotein E, linked to a model class A amphipathic helix, enhances internalization and degradation of LDL by fibroblasts. Biochemistry.

[78] Spencer B.J., Verma I.M. (2007). Targeted delivery of proteins across the blood-brain barrier. Proc. Natl. Acad. Sci. USA.

[79] Wang D., El-Amouri S.S., Dai M., Kuan C.Y., Hui D.Y., Brady R.O., Pan D. (2013). Engineering a lysosomal enzyme with a derivative of receptor-binding domain of apoE enables delivery across the blood-brain barrier. Proc. Natl. Acad. Sci. USA.

[80] Tian T., Zhang H.X., He C.P., Fan S., Zhu Y.L., Qi C., Huang N.P., Xiao Z.D., Lu Z.H., Tannous B.A. (2018). Surface functionalized exosomes as targeted drug delivery vehicles for cerebral ischemia therapy. Biomaterials.

[81] Kumar P., Wu H., McBride J.L., Jung K.E., Kim M.H., Davidson B.L., Lee S.K., Shankar P., Manjunath N. (2007). Transvascular delivery of small interfering RNA to the central nervous system. Nature.

[82] Javed H., Menon S.A., Al-Mansoori K.M., Al-Wandi A., Majbour N.K., Ardah M.T., Varghese S., Vaikath N.N., Haque M.E., Azzouz M. (2016). Development of Nonviral Vectors Targeting the Brain as a Therapeutic Approach for Parkinson’s Disease and Other Brain Disorders. Mol. Ther..

[83] Lee J.H., Zhang A., You S.S., Lieber C.M. (2016). Spontaneous Internalization of Cell Penetrating Peptide-Modified Nanowires into Primary Neurons. Nano Lett..

[84] Neves V., Aires-da-Silva F., Morais M., Gano L., Ribeiro E., Pinto A., Aguiar S., Gaspar D., Fernandes C., Correia J.D.G. (2017). Novel Peptides Derived from Dengue Virus Capsid Protein Translocate Reversibly the Blood-Brain Barrier through a Receptor-Free Mechanism. ACS Chem. Biol..

[85] Oller-Salvia B., Sánchez-Navarro M., Ciudad S., Guiu M., Arranz-Gibert P., Garcia C., Gomis R.R., Cecchelli R., García J., Giralt E. (2016). MiniAp-4: A Venom-Inspired Peptidomimetic for Brain Delivery. Angew. Chem. Int. Ed. Engl..

[86] Wu C.H., Liu I.J., Lu R.M., Wu H.C. (2016). Advancement and applications of peptide phage display technology in biomedical science. J. Biomed. Sci..

[87] Gao H., Qian J., Cao S., Yang Z., Pang Z., Pan S., Fan L., Xi Z., Jiang X., Zhang Q. (2012). Precise glioma targeting of and penetration by aptamer and peptide dual-functioned nanoparticles. Biomaterials.

[88] Majerova P., Hanes J., Olesova D., Sinsky J., Pilipcinec E., Kovac A. (2020). Novel blood–brain barrier shuttle peptides discovered through the phage display method. Molecules.

[89] Torchilin V.P. (2006). Recent approaches to intracellular delivery of drugs and DNA and organelle targeting. Annu. Rev. Biomed. Eng..

[90] Liu L., Guo K., Lu J., Venkatraman S.S., Luo D., Ng K.C., Ling E.A., Moochhala S., Yang Y.Y. (2008). Biologically active core/shell nanoparticles self-assembled from cholesterol-terminated PEG-TAT for drug delivery across the blood-brain barrier. Biomaterials.

[91] Agyare E.K., Curran G.L., Ramakrishnan M., Yu C.C., Poduslo J.F., Kandimalla K.K. (2008). Development of a smart nano-vehicle to target cerebrovascular amyloid deposits and brain parenchymal plaques observed in Alzheimer’s disease and cerebral amyloid angiopathy. Pharm. Res..

[92] Dong X. (2018). Current Strategies for Brain Drug Delivery. Theranostics.

[93] Sharma G., Modgil A., Layek B., Arora K., Sun C., Law B., Singh J. (2013). Cell penetrating peptide tethered bi-ligand liposomes for delivery to brain in vivo: Biodistribution and transfection. J. Control. Release.

[94] Urbiola K., Blanco-Fernández L., Ogris M., Rödl W., Wagner E., Tros de Ilarduya C. (2018). Novel PAMAM-PEG-Peptide Conjugates for siRNA Delivery Targeted to the Transferrin and Epidermal Growth Factor Receptors. Pers. Med..

[95] Lu F., Pang Z., Zhao J., Jin K., Li H., Pang Q., Zhang L., Pang Z. (2017). Angiopep-2-conjugated poly(ethylene glycol)-co- poly(ε-caprolactone) polymersomes for dual-targeting drug delivery to glioma in rats. Int. J. Nanomed..

[96] Ahlschwede K.M., Curran G.L., Rosenberg J.T., Grant S.C., Sarkar G., Jenkins R.B., Ramakrishnan S., Poduslo J.F., Kandimalla K.K. (2019). Cationic carrier peptide enhances cerebrovascular targeting of nanoparticles in Alzheimer’s disease brain. Nanomedicine.

[97] Nosrati H., Tarantash M., Bochani S., Charmi J., Bagheri Z., Fridoni M., Abdollahifar M.A., Davaran S., Danafar H., Kheiri Manjili H. (2019). Glutathione (GSH) peptide conjugated magnetic nanoparticles as blood–brain barrier shuttle for MRI-monitored brain delivery of paclitaxel. ACS Biomater. Sci. Eng..

[98] Wang S., Zhang B., Su L., Nie W., Han D., Han G., Zhang H., Chong C., Tan J. (2019). Subcellular distributions of iron oxide nanoparticles in rat brains affected by different surface modifications. Biomed. Mater. Res. A.

[99] Yang L., Qian W., Scott P., Shao X. (2018). Towards the development of brain-penetrating gold nanoparticle-transactivator of transcription (TAT) peptide conjugates. J. Nucl. Med..

[100] Kang J., Joo J., Kwon E.J., Skalak M., Hussain S., She Z.G., Ruoslahti E., Bhatia S.N., Sailor M.J. (2016). Self-Sealing Porous Silicon-Calcium Silicate Core-Shell Nanoparticles for Targeted siRNA Delivery to the Injured Brain. Adv. Mater..

[101] Haney M.J., Klyachko N.L., Zhao Y., Gupta R., Plotnikova E.G., He Z., Patel T., Piroyan A., Sokolsky M., Kabanov A.V. (2015). Exosomes as drug delivery vehicles for Parkinson’s disease therapy. J. Control. Release.

[102] Luan X., Sansanaphongpricha K., Myers I., Chen H., Yuan H., Sun D. (2017). Engineering exosomes as refined biological nanoplatforms for drug delivery. Acta Pharmacol. Sin..

[103] Alvarez-Erviti L., Seow Y., Yin H., Betts C., Lakhal S., Wood M.J. (2011). Delivery of siRNA to the mouse brain by systemic injection of targeted exosomes. Nat. Biotechnol..

[104] Pusic A.D., Pusic K.M., Clayton B.L., Kraig R.P. (2014). IFNγ-stimulated dendritic cell exosomes as a potential therapeutic for remyelination. J. Neuroimmunol..

[105] Islam Y., Leach A.G., Smith J., Pluchino S., Coxonl C.R., Sivakumaran M., Downing J., Fatokun A.A., Teixidò M., Ehtezazi T. (2020). Peptide based drug delivery systems to the brain. Nano. Express.

[106] Cooper J.M., Wiklander P.B., Nordin J.Z., Al-Shawi R., Wood M.J., Vithlani M., Schapira A.H., Simons J.P., El-Andaloussi S., Alvarez-Erviti L. (2014). Systemic exosomal siRNA delivery reduced alpha-synuclein aggregates in brains of transgenic mice. Mov. Disord..

[107] Pulford B., Reim N., Bell A., Veatch J., Forster G., Bender H., Meyerett C., Hafeman S., Michel B., Johnson T. (2010). Liposome-siRNA-peptide complexes cross the blood-brain barrier and significantly decrease PrP on neuronal cells and PrP in infected cell cultures. PLoS ONE.

[108] Bender H.R., Kane S., Zabel M.D. (2016). Delivery of Therapeutic siRNA to the CNS Using Cationic and Anionic Liposomes. J. Vis. Exp..

[109] Grinberg S., Linder C., Kolot V., Waner T., Wiesman Z., Shaubi E., Heldman E. (2005). Novel cationic amphiphilic derivatives from vernonia oil: Synthesis and self-aggregation into bilayer vesicles, nanoparticles, and DNA complexants. Langmuir.

[110] Popov M., Abu Hammad I., Bachar T., Grinberg S., Linder C., Stepensky D., Heldman E. (2013). Delivery of analgesic peptides to the brain by nano-sized bolaamphiphilic vesicles made of monolayer membranes. Eur. J. Pharm. Biopharm..

[111] Conceição M., Mendonça L., Nóbrega C., Gomes C., Costa P., Hirai H., Moreira J.N., Lima M.C., Manjunath N., Pereira de Almeida L. (2016). Intravenous administration of brain-targeted stable nucleic acid lipid particles alleviates Machado-Joseph disease neurological phenotype. Biomaterials.

[112] Lu M., Xing H., Xun Z., Yang T., Zhao X., Cai C., Wang D., Ding P. (2018). Functionalized extracellular vesicles as advanced therapeutic nanodelivery systems. Eur. J. Pharm. Sci..

[113] Ke W., Shao K., Huang R., Han L., Liu Y., Li J., Kuang Y., Ye L., Lou J., Jiang C. (2009). Gene delivery targeted to the brain using an Angiopep-conjugated polyethyleneglycol-modified polyamidoamine dendrimer. Biomaterials.

[114] Perez A.P., Romero E.L., Morilla M.J. (2009). Ethylendiamine core PAMAM dendrimers/siRNA complexes as in vitro silencing agents. Int. J. Pharm..

[115] Al-Azzawi S., Masheta D., Guildford A., Phillips G., Santin M. (2019). Designing and Characterization of a Novel Delivery System for Improved Cellular Uptake by Brain Using Dendronised Apo-E-Derived Peptide. Front. Bioeng. Biotechnol..

[116] Zhou X., Smith Q.R., Liu X. (2021). Brain penetrating peptides and peptide-drug conjugates to overcome the blood-brain barrier and target CNS diseases. Wiley Interdiscip. Rev. Nanomed. Nanobiotechnol..

[117] Zhang H., Gerson T., Varney M.L., Singh R.K., Vinogradov S.V. (2010). Multifunctional peptide-PEG intercalating conjugates: Programmatic of gene delivery to the blood-brain barrier. Pharm. Res..

[118] Sun R., Liu M., Lu J., Chu B., Yang Y., Song B., Wang H., He Y. (2022). Bacteria loaded with glucose polymer and photosensitive ICG silicon-nanoparticles for glioblastoma photothermal immunotherapy. Nat. Commun..

[119] Wu L.P., Ahmadvand D., Su J., Hall A., Tan X., Farhangrazi Z.S., Moghimi S.M. (2019). Crossing the blood-brain-barrier with nanoligand drug carriers self-assembled from a phage display peptide. Nat. Commun..

[120] Smith T.L., Sidman R.L., Arap W., Pasqualini R. (2022). Targeting vascular zip codes: From combinatorial selection to drug prototypes. The Vasculome.

[121] Cho C.F., Farquhar C.E., Fadzen C.M., Scott B., Zhuang P., von Spreckelsen N., Loas A., Hartrampf N., Pentelute B.L., Lawler S.E. (2022). A Tumour-Homing Peptide Platform Enhances Drug Solubility, Improves Blood-Brain Barrier Permeability and Targets Glioblastoma. Cancers.

[122] Eriste E., Kurrikoff K., Suhorutšenko J., Oskolkov N., Copolovici D.M., Jones S., Laakkonen P., Howl J., Langel Ü. (2013). Peptide-based glioma-targeted drug delivery vector gHoPe2. Bioconjug. Chem..

[123] Prades R., Oller-Salvia B., Schwarzmaier S.M., Selva J., Moros M., Balbi M., Grazú V., de La Fuente J.M., Egea G., Plesnila N. (2015). Applying the retro-enantio approach to obtain a peptide capable of overcoming the blood-brain barrier. Angew. Chem. Int. Ed. Engl..

[124] Prades R., Guerrero S., Araya E., Molina C., Salas E., Zurita E., Selva J., Egea G., López-Iglesias C., Teixidó M. (2012). Delivery of gold nanoparticles to the brain by conjugation with a peptide that recognizes the transferrin receptor. Biomaterials.

[125] Zou Z., Shen Q., Pang Y., Li X., Chen Y., Wang X., Luo X., Wu Z., Bao Z., Zhang J. (2019). The synthesized transporter K16APoE enabled the therapeutic HAYED peptide to cross the blood-brain barrier and remove excess iron and radicals in the brain, thus easing Alzheimer’s disease. Drug Deliv. Transl. Res..

[126] Tosi G., Badiali L., Ruozi B., Vergoni A.V., Bondioli L., Ferrari A., Rivasi F., Forni F., Vandelli M.A. (2012). Can leptin-derived sequence-modified nanoparticles be suitable tools for brain delivery?. Nanomedicine.

[127] Zhuang X., Xiang X., Grizzle W., Sun D., Zhang S., Axtell R.C., Ju S., Mu J., Zhang L., Steinman L. (2011). Treatment of brain inflammatory diseases by delivering exosome encapsulated anti-inflammatory drugs from the nasal region to the brain. Mol. Ther..

[128] Napoli C., Lemieux C., Jorgensen R. (1990). Introduction of a chimeric chalcone synthase gene into petunia results in reversible co-suppression of homologous genes in trans. Plant Cell.

[129] Tatiparti K., Sau S., Kashaw S.K., Iyer A.K. (2017). siRNA delivery strategies: A comprehensive review of recent developments. Nanomaterials.

[130] Xie X., Lin W., Li M., Yang Y., Deng J., Liu H., Chen Y., Fu X., Liu H., Yang Y. (2016). Efficient siRNA delivery using novel cell-penetrating peptide-siRNA conjugate-loaded nanobubbles and ultrasound. Ultrasound. Med. Biol..

[131] Pratt A.J., MacRae I.J. (2009). The RNA-induced silencing complex: A versatile gene-silencing machine. J. Biol. Chem..

[132] Mathupala S.P. (2009). Delivery of small-interfering RNA (siRNA) to the brain. Expert. Opin. Ther. Pat..

[133] Yu Z., Ye J., Pei X., Sun L., Liu E., Wang J., Huang Y., Lee S.J., He H. (2018). Improved method for synthesis of low molecular weight protamine–siRNA conjugate. Acta Pharm. Sin. B.

[134] Pärnaste L., Arukuusk P., Langel K., Tenson T., Langel Ü. (2017). The Formation of Nanoparticles between Small Interfering RNA and Amphipathic Cell-Penetrating Peptides. Mol. Ther. Nucleic Acids.

[135] Matsushita-Ishiodori Y., Ohtsuki T. (2012). Photoinduced RNA interference. Acc. Chem. Res..

[136] Zeller S., Choi C.S., Uchil P.D., Ban H.-S., Siefert A., Fahmy T.M., Mothes W., Lee S.-K., Kumar P. (2015). Attachment of cell-binding ligands to arginine-rich cell-penetrating peptides enables cytosolic translocation of complexed siRNA. Chem. Biol..

[137] Youn P., Chen Y., Furgeson D.Y. (2014). A myristoylated cell-penetrating peptide bearing a transferrin receptor-targeting sequence for neuro-targeted siRNA delivery. Mol. Pharm..

[138] Xu Y.Y., Cao X.W., Fu L.Y., Zhang T.Z., Wang F.J., Zhao J. (2019). Screening and characterization of a novel high-efficiency tumour-homing cell-penetrating peptide from the buffalo cathelicidin family. J. Pept. Sci..

[139] Kang J.H., Battogtokh G., Ko Y.T. (2017). Self-assembling lipid–peptide hybrid nanoparticles of phospholipid–nonaarginine conjugates for enhanced delivery of nucleic acid therapeutics. Biomacromolecules.

[140] Hatakeyama H., Akita H., Kogure K., Oishi M., Nagasaki Y., Kihira Y., Ueno M., Kobayashi H., Kikuchi H., Harashima H. (2007). Development of a novel systemic gene delivery system for cancer therapy with a tumour-specific cleavable PEG-lipid. Gene Ther..

[141] Xiang B., Jia X.-L., Qi J.-L., Yang L.-P., Sun W.-H., Yan X., Yang S.-K., Cao D.-Y., Du Q., Qi X.-R. (2017). Enhancing siRNA-based cancer therapy using a new pH-responsive activatable cell-penetrating peptide-modified liposomal system. Int. J. Nanomed..

[142] Yang Y., Xie X., Yang Y., Li Z., Yu F., Gong W., Li Y., Zhang H., Wang Z., Mei X. (2016). Polymer nanoparticles modified with photo-and pH-dual-responsive polypeptides for enhanced and targeted cancer therapy. Mol. Pharm..

[143] Richard C.M. (1993). The basic science of gene therapy. Science.

[144] Quinonez R., Sutton R.E. (2002). Lentiviral vectors for gene delivery into cells. DNA Cell Biol..

[145] Zabel M.D., Mollnow L., Bender H. (2021). siRNA Therapeutics for Protein Misfolding Diseases of the Central Nervous System. Des. Deliv. SiRNA Ther..

[146] Chiu Y.L., Ali A., Chu C.Y., Cao H., Rana T.M. (2004). Visualizing a correlation between siRNA localization, cellular uptake, and RNAi in living cells. Chem. Biol..

[147] Meade B.R., Dowdy S.F. (2008). Enhancing the cellular uptake of siRNA duplexes following noncovalent packaging with protein transduction domain peptides. Adv. Drug. Deliv. Rev..

[148] Barichello J.M., Ishida T., Kiwada H. (2010). Complexation of siRNA and pDNA with cationic liposomes: The important aspects in lipoplex preparation. Liposomes Methods Protoc. Pharm. Nanocarriers.

[149] Malone R.W., Felgner P.L., Verma I.M. (1989). Cationic liposome-mediated RNA transfection. Proc. Natl. Acad. Sci. USA.

[150] Balazs D.A., Godbey W. (2011). Liposomes for use in gene delivery. J. Drug Deliv..

[151] Patil S.D., Burgess D.J. (2003). DNA-based Biopharmaceuticals: Therapeutics for the 21st Century. AAPS Newsmag..

[152] Georgieva J.V., Hoekstra D., Zuhorn I.S. (2014). Smuggling drugs into the brain: An overview of ligands targeting transcytosis for drug delivery across the blood–brain barrier. Pharmaceutics.

[153] Uno Y., Piao W., Miyata K., Nishina K., Mizusawa H., Yokota T. (2011). High-density lipoprotein facilitates in vivo delivery of α-tocopherol–conjugated short-interfering RNA to the brain. Hum. Gene Ther..

[154] Papahadjopoulos D., Allen T., Gabizon A., Mayhew E., Matthay K., Huang S., Lee K., Woodle M., Lasic D., Redemann C. (1991). Sterically stabilized liposomes: Improvements in pharmacokinetics and antitumour therapeutic efficacy. Proc. Natl. Acad. Sci. USA.

[155] Zamboni W.C., Ramalingam S., Friedland D.M., Edwards R.P., Stoller R.G., Strychor S., Maruca L., Zamboni B.A., Belani C.P., Ramanathan R.K. (2009). Phase I and pharmacokinetic study of pegylated liposomal CKD-602 in patients with advanced malignancies. Clin. Cancer Res..

[156] Battaglia L., Panciani P.P., Muntoni E., Capucchio M.T., Biasibetti E., De Bonis P., Mioletti S., Fontanella M., Swaminathan S. (2018). Lipid nanoparticles for intranasal administration: Application to nose-to-brain delivery. Expert Opin. Drug Deliv..

[157] Erdő F., Bors L.A., Farkas D., Bajza Á., Gizurarson S. (2018). Evaluation of intranasal delivery route of drug administration for brain targeting. Brain Res. Bull..

[158] Danielyan L., Schäfer R., von Ameln-Mayerhofer A., Buadze M., Geisler J., Klopfer T., Burkhardt U., Proksch B., Verleysdonk S., Ayturan M. (2009). Intranasal delivery of cells to the brain. Eur. J. Cell Biol..

[159] Danielyan L., Schäfer R., von Ameln-Mayerhofer A., Bernhard F., Verleysdonk S., Buadze M., Lourhmati A., Klopfer T., Schaumann F., Schmid B. (2011). Therapeutic efficacy of intranasally delivered mesenchymal stem cells in a rat model of Parkinson disease. Rejuvenation Res..

[160] Danielyan L., Beer-Hammer S., Stolzing A., Schäfer R., Siegel G., Fabian C., Kahle P., Biedermann T., Lourhmati A., Buadze M. (2014). Intranasal delivery of bone marrow-derived mesenchymal stem cells, macrophages, and microglia to the brain in mouse models of Alzheimer’s and Parkinson’s disease. Cell Transplant..

[161] Donega V., Nijboer C.H., van Tilborg G., Dijkhuizen R.M., Kavelaars A., Heijnen C.J. (2014). Intranasally administered mesenchymal stem cells promote a regenerative niche for repair of neonatal ischemic brain injury. Exp. Neurol..

[162] Oppliger B., Joerger-Messerli M., Mueller M., Reinhart U., Schneider P., Surbek D.V., Schoeberlein A. (2016). Intranasal delivery of umbilical cord-derived mesenchymal stem cells preserves myelination in perinatal brain damage. Stem Cells Dev..

[163] Balyasnikova I.V., Prasol M.S., Ferguson S.D., Han Y., Ahmed A.U., Gutova M., Tobias A.L., Mustafi D., Rincón E., Zhang L. (2014). Intranasal delivery of mesenchymal stem cells significantly extends survival of irradiated mice with experimental brain tumours. Mol. Ther..

[164] Raposo G., Stoorvogel W. (2013). Extracellular vesicles: Exosomes, microvesicles, and friends. J. Cell Biol..

[165] Valadi H., Ekström K., Bossios A., Sjöstrand M., Lee J.J., Lötvall J.O. (2007). Exosome-mediated transfer of mRNAs and microRNAs is a novel mechanism of genetic exchange between cells. Nat. Cell Biol..

[166] Izco M., Carlos E., Alvarez-Erviti L. (2022). The two faces of Exosomes in Parkinson’s disease: From pathology to therapy. Neuroscientist.

[167] Cheng Z., Al Zaki A., Hui J.Z., Muzykantov V.R., Tsourkas A. (2012). Multifunctional nanoparticles: Cost versus benefit of adding targeting and imaging capabilities. Science.

[168] Torchilin V.P. (2014). Multifunctional, stimuli-sensitive nanoparticulate systems for drug delivery. Nat. Rev. Drug Discov..

[169] Castanotto D., Rossi J.J. (2009). The promises and pitfalls of RNA-interference-based therapeutics. Nature.

